# Associations of health-related quality of life with major adverse cardiovascular and cerebrovascular events for individuals with ischaemic heart disease: systematic review, meta-analysis and evidence mapping

**DOI:** 10.1136/openhrt-2023-002452

**Published:** 2023-10-27

**Authors:** Anzhela Soloveva, Chris P Gale, Han Naung Tun, Ben Hurdus, Suleman Aktaa, Victoria Palin, Teumzghi F Mebrahtu, Harriette Van Spall, Gorav Batra, Tatendashe Bernadette Dondo, Maria Bäck, Theresa Munyombwe

**Affiliations:** 1 Department of Cardiology, Almazov National Medical Research Centre, Sankt-Peterburg, Russian Federation; 2 Leeds Institute of Cardiovascular and Metabolic Medicine/Leeds Institute of Data analytics, University of Leeds, Leeds, UK; 3 Larner College of Medicine, University of Vermont, Burlington, Vermont, USA; 4 Department of Cardiology, Leeds General Infirmary, Leeds, UK; 5 Bradford Institute for Health Research, Bradford Teaching Hospitals NHS Foundation Trust, Bradford, UK; 6 Population Health Research Institute, Hamilton, Ontario, Canada; 7 Division of Cardiology, McMaster University, Hamilton, Ontario, Canada; 8 Cardiology and Uppsala Clinical Research Center, Uppsala University, Uppsala, Sweden; 9 Department of Medical and Health Sciences, Linköping University, Linkoping, Sweden

**Keywords:** health-related quality of life, cardiovascular outcomes, mortality, systematic review, meta-analysis

## Abstract

**Objective:**

To investigate the association between health-related quality of life (HRQoL) and major adverse cardiovascular and cerebrovascular events (MACCE) in individuals with ischaemic heart disease (IHD).

**Methods:**

Medline(R), Embase, APA PsycINFO and CINAHL (EBSCO) from inception to 3 April 2023 were searched. Studies reporting association of HRQoL, using a generic or cardiac-specific tool, with MACCE or components of MACCE for individuals with IHD were eligible for inclusion. Risk of bias was assessed using the Newcastle-Ottawa Quality Assessment Scale to assess the quality of the studies. Descriptive synthesis, evidence mapping and random-effects meta-analysis were performed stratified by HRQoL measures and effect estimates. Between-study heterogeneity was assessed using the Higgins I^2^ statistic.

**Results:**

Fifty-one articles were included with a total of 134 740 participants from 53 countries. Meta-analysis of 23 studies found that the risk of MACCE increased with lower baseline HeartQoL score (HR 1.49, 95% CI 1.16 to 1.93) and Short Form Survey (SF-12) physical component score (PCS) (HR 1.39, 95% CI 1.28 to 1.51). Risk of all-cause mortality increased with a lower HeartQoL (HR 1.64, 95% CI 1.34 to 2.01), EuroQol 5-dimension (HR 1.17, 95% CI 1.12 to 1.22), SF-36 PCS (HR 1.29, 95% CI 1.19 to 1.41), SF-36 mental component score (HR 1.18, 95% CI 1.08 to 1.30).

**Conclusions:**

This study found an inverse association between baseline values or change in HRQoL and MACCE or components of MACCE in individuals with IHD, albeit with between-study heterogeneity. Standardisation and routine assessment of HRQoL in clinical practice may help risk stratify individuals with IHD for tailored interventions.

**PROSPERO registration number:**

CRD42021234638.

**What is already known on this topic**

- Health-related quality of life is a key outcome in people with ischaemic heart disease.
- The association between poor health-related quality of life and adverse clinical outcomes in patients with ischaemic heart disease remains unclear.
- Systematic reviews and meta-analyses of the association between health-related quality of life and major adverse cardiac and cerebrovascular events in individuals with ischaemic heart disease are lacking.

**What this study adds**

- This systematic review and meta-analysis found that poor health-related quality of life at baseline and its deterioration over time was associated with an increased risk of major adverse cardiac and cerebrovascular events in individuals with ischaemic heart disease.
- The inverse association between health-related quality of life and clinical outcomes in individuals with ischaemic heart disease was evident across a range of patient-reported outcomes measures and types of ischaemic heart disease.

**How this study might affect research, practice or policy**

- There is between-study heterogeneity with respect to health-related quality of life instruments used, population characteristics, follow-up durations and data analysis methods creating a need to standardise the approach by which health-related quality of life is assessed in individuals with ischaemic heart disease.
- Routine capture of health-related quality of life in clinical practice may be an opportunity to monitor and risk stratify individuals with ischaemic heart disease.

## Introduction

Health-related quality of life (HRQoL) is a key outcome in cardiovascular disease. Historically, healthcare professionals have focused on objective measures of health, such as mortality and life expectancy, but patients consider improvements in their HRQoL equally important as their length of life.^1^ Traditional measures of outcomes including mortality and hospitalisation insufficiently capture the benefits of medical interventions for chronic disease states such as ischaemic heart disease (IHD) and do not reflect symptom burden, physical function, psychological well-being and social interaction.^2 3^ Moreover, there is a discord between patients’ and physicians’ evaluations of successful outcomes after clinical interventions.^4 5^ Accordingly, many studies now include patient-reported outcome measures to evaluate the impact of care on patient health status,^6 7^ and most of the recent guidelines promote HRQoL assessment as a complementary health outcome measure, highlighting a shift towards patient-centeredness of cardiovascular care.^8 9^

For cardiovascular disease, lower patient-reported HRQoL is associated with increased mortality,^10^ and may be a prognostic indicator of cardiac end points, such that it could be used to identify individuals in need of tailored interventions. For IHD, however, there is limited information about the association between HRQoL and cardiovascular events. In 2009, a systematic review suggested that HRQoL in patients with coronary artery disease was inversely associated with mortality and hospitalisation.^11^ However, this was not accompanied by a meta-analysis. While a number of studies have been published since showing similar findings,^12–14^ there remains uncertainty about this association.^15^ A systematic review that included a meta-analysis^16^ focused on the association of current self-rated health measured by a single question at the beginning of follow-up. This study did not consider domains of HRQoL or validated measures. Our systematic review expanded this work by considering studies that used validated HRQoL measures and their domains. It is important to consider HRQoL domains to identify the specific components of HRQoL that need targeted interventions.

We, therefore, conducted a systematic review with meta-analysis and evidence mapping to examine the associations of overall HRQoL and domains with major adverse cardiac and cerebrovascular events (MACCE) in patients with IHD.

## Methods

### Protocol and guidance

Our systematic review was reported in accordance with the 2020 Preferred Reporting Items for Systematic Reviews and Meta-Analyses (PRISMA) statement^17^ (online supplemental material 1). The review protocol was registered and further updated at PROSPERO (CRD42021234638, update in March 2023). Differences between the versions of the protocol are explained (online supplemental material 2).

10.1136/openhrt-2023-002452.supp1 Supplementary data

### Eligibility criteria

Studies were eligible if they analysed associations of HRQoL using any generic or cardiac-specific tools (online supplemental material 1), with MACCE or components of MACCE for individuals with IHD. The prespecified primary end point was MACCE, comprising death (all-cause death, cardiovascular death), myocardial infarction (MI), stroke, heart failure hospitalisation, hospitalisation with unstable angina or coronary revascularisation (percutaneous coronary intervention (PCI), coronary artery bypass graft (CABG) surgery). Studies reporting any of the components of the primary end point were included in the analysis. No language, study sample or date restrictions were applied.

### Search strategy

We analysed all studies included in the previously published systematic review^11^ and conducted electronic searches for eligible studies within Medline(R), Embase Classic and Embase, APA PsycINFO and CINAHL (EBSCO) databases from inception until 3 April 2023. The search strategy (online supplemental material 4) included the following concepts: conditions or procedures linked to IHD, quality of life, predictive factors and MACCE, hospital readmission or mortality. Given that HRQoL is a subjective and multidimensional concept and to increase the search sensitivity, we did not include specific domain terms, but included names of the selected generic and cardiac-specific instruments (online supplemental material 3).

### Study selection

Using the Rayyan website, two authors (VP, NTH) independently screened titles and abstracts for agreed inclusion criteria. Disagreements were resolved by consensus (VP, NTH, AS). Following this, full-text articles were retrieved and assessed for eligibility (AS). Attempts to obtain full-text manuscripts of non-retrieved articles by contacts with authors were undertaken.

### Data extraction

For each article, publication characteristics (first author, year of publication), study characteristics (name of the study, country, recruitment period, inclusion and exclusion criteria, enrolment approach, sample size, longest time of patient follow-up), patients’ characteristics (age, sex, race, cardiovascular risk factors and comorbidities, proportions of patients with prior and index MI, PCI, CABG surgery), HRQoL instruments used (including domains, and assessment features—settings, time and frequency), outcomes and data sources used for outcomes collection were extracted into a predesigned form. Effect or risk measures extracted were HRs, ORs and incidence rate ratios with their corresponding 95% CIs or other estimates of associations between HRQoL and outcome measures. The first reviewer (AS) completed the data extraction form and second reviewers (TM, VP, NTH) verified the extracted information. In case of missing or unclear data, additional information was searched through previously published protocols and/or primary results of an appropriate study.

### Risk of bias (quality) assessment

The Newcastle-Ottawa Quality Assessment Scale was used to assess the risk of bias and overall quality of the included studies.^18^ The methodological domains (selection, comparability and outcome assessment) were rated by one reviewer (AS) and independently verified by a second reviewer (TM). For each of the included articles, the results were summarised into overall judgement on good, fair or poor study quality (online supplemental material 5).

### Data synthesis and statistical analysis

Evidence synthesis was conducted using qualitative approaches, evidence mapping and meta-analysis. All included articles were divided into those reporting associations of HRQoL with all-cause mortality, MACCE and a component of MACCE. The data synthesis on the predictive value of HRQoL estimates were mapped, summarising by the year of publication, sample size, nosological form of IHD, HRQoL questionnaire.

Effect estimates for HRQoL with different unit increments and (or) directionality were standardised through mathematical transformations described in online supplemental material 6.

Due to the heterogeneity across the studies, stratified meta-analysis was conducted by grouping studies that used the same HRQoL measure and reported comparable effect estimates (either HR (incidence rate ratios) or OR). A random-effects meta-analysis was conducted using the metan package in Stata.^19^ Heterogeneity across the studies was assessed using the Higgins I^2^ statistic and (I^2^ >50% suggested substantial heterogeneity).^20^ The effect sizes and their 95% CIs were displayed in forest plots. Data analysis was undertaken using Stata statistical software, V.16.1 (StataCorp, College Station, Texas, USA).

### Certainty assessment

The Grading of Recommendations, Assessment, Development and Evaluations (GRADE) approach was used to assess the certainty of evidence for each outcome (AS, NTH). For each MACCE outcome, the low certainty of the evidence that is attributable to the observational studies was downgraded into very low (based on high risk of bias, inconsistency of effect, indirectness, imprecision, publication bias) or upgraded into low, moderate and high (based on the effect size, dose-response gradient and adjustment for confounding factors).^21^

## Results

### Study selection

**Figure 1:**
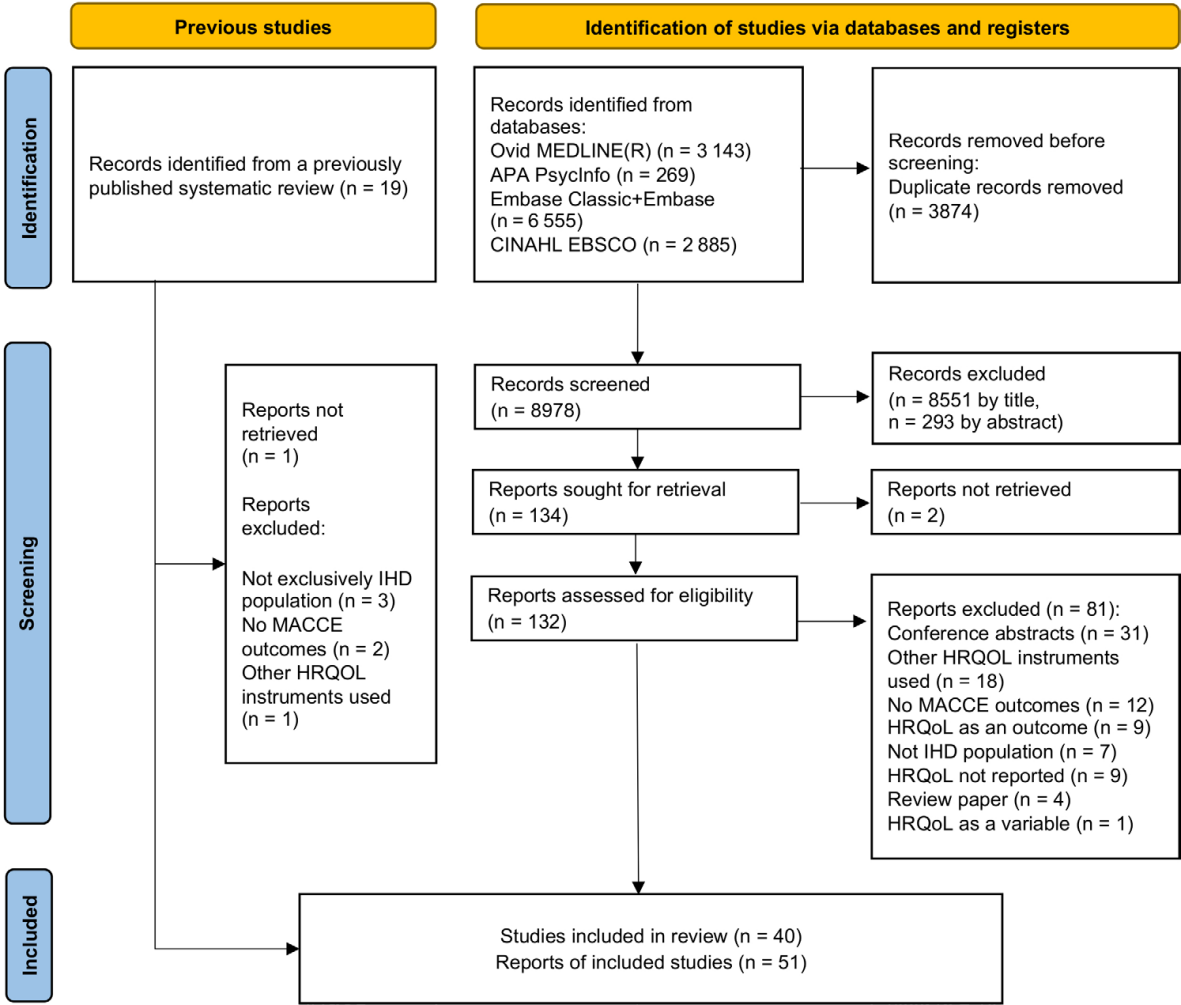
Preferred Reporting Items for Systematic Reviews and Meta-Analyses flow chart. HRQoL, health-related quality of life;IHD, ischaemic heart disease; MACCE, major adverse cardiac and cerebrovascular events.

Of the 8978 studies initially identified as potentially addressing the associations of HRQoL with MACCE in IHD, 8844 were excluded after title/abstract screening. Of 132 articles that underwent full-text screening, 50 met the inclusion criteria (figure 1). One article not captured by the search strategy^22^ was included manually based on the analysis of previously published systematic review^11^ (online supplemental table S1).

### Study characteristics

**Figure 2:**
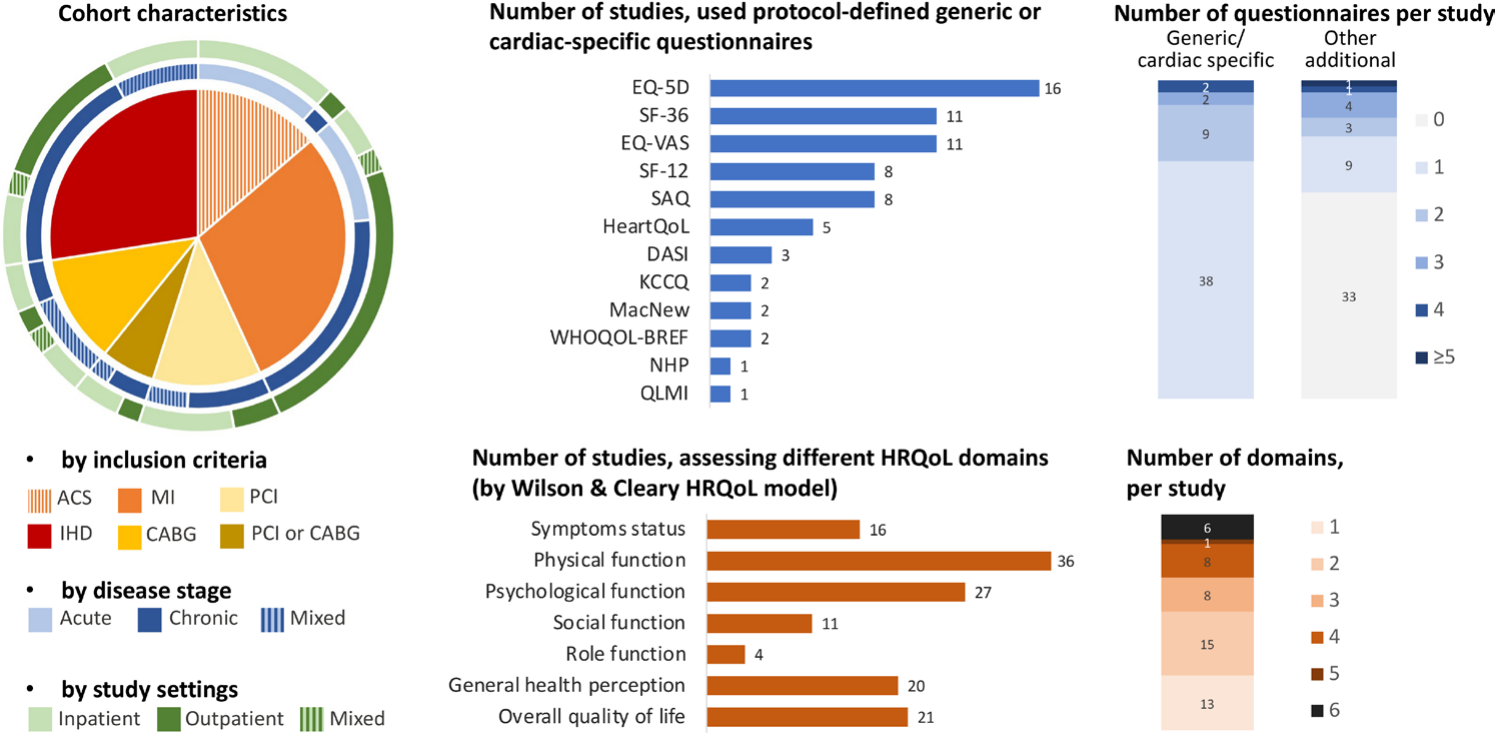
Characteristics of included studies. ACS, acute coronary syndrome; CABG, coronary artery bypass graft; DASI, Duke Activity Status Index; EQ-5D, EuroQol 5-dimension; EQ-VAS, EuroQol visual analogue scale; HRQoL, health-related quality of life; IHD, ischaemic heart disease; KCCQ, The Kansas City Cardiomyopathy Questionnaire; MacNew, MacNew Questionnaire; MI, myocardial infarction; NHP, Nottingham Health Profile; PCI, percutaneous coronary intervention; QLMI, Quality of Life after Myocardial Infarction; SAQ, Seattle Angina Questionnaire; SF-12, 12-Item Short Form Survey; SF-36, 36-Item Short Form Survey; WHOQOL-BREF, WHO Quality of Life Questionnaire, brief version.

The main characteristics of included studies are shown in table 1 and figure 2. There were 51 articles reporting 40 unique cohorts with a total of 134 740 participants from 53 countries. In total, 23 (57.5%) cohorts were multicentre observational studies, 4 (10%) were post hoc analyses of randomised controlled trials and 13 (32.5%) were single-centre studies. All studies were conducted in high-income countries except five multicentre international studies that also included participants from upper-middle-income^13 14 23–25^ and lower-middle-income countries^24^ and one single-centre study from lower-middle-income country.^26^

**Table 1:** Characteristics of included publications

Characteristic	All publications included (n=51)	All-cause mortality (n=35, 68.6%)	Cardiovascular and vascular mortality (n=7, 13.7%)	MACCE	Component of MACCE
				(n=25, 49.0%)	(n=6, 11.8%)
Main inclusion criteria					
ACS	7 (13.7%)	3 (8.6%)	–	4 (6.0%)	–
MI	15 (29.4%)	9 (25.7%)	2 (28.6%)	10 (40.0%)	2 (33.3%)
PCI and/or CABG	14 (27.5%)	11 (31.4%)	3 (42.9%)	6 (24.0%)	2 (33.3%)
IHD	15 (29.4%)	12 (34.3%)	2 (28.6%)	5 (20.0%)	2 (33.3%)
Cohort					
Total sample size					
<100	1 (1.9 %)	–	–	1 (4.0%)	–
100–499	9 (17.6%)	3 (8.6%)	1 (14.3%)	7 (28.0%)	1 (16.7%)
500–999	11 (21.6%)	7 (20.0%)	–	7 (28.0%)	–
1000–4999	19 (37.3%)	19 (54.3%)	4 (57.1%)	4 (6.0%)	5 (83.3%)
≥5000	11 (21.6%)	6 (17.1%)	2 (28.6%)	6 (24.0%)	–
Mean age ≤65 years	34 (66.7%)	24 (68.6%)	5 (71.4%)	12 (48.0%)	3 (50.0%)
Proportion of female					
≤30%	38 (74.5%)	26 (68.6%)	6 (85.7%)	17 (68.0%)	5 (83.3%)
>30%	10 (19.6%)	7 (20.0%)	1 (14.3%)	6 (24.0%)	1 (16.7%)
100%	3 (5.9%)	2 (5.7%)	–	2 (8.0%)	–
Race					
Not reported	38 (74.5%)	25 (71.4%)	4 (57.1%)	20 (80.0%)	3 (50%)
White <80%	3 (5.9%)	2 (5.7%)	1 (14.3%)	1 (4.0%)	1 (16.7%)
Lost to follow-up					
≤20% of participants	36 (70.6%)	26 (74.3%)	3 (42.9%)	18 (72.0%)	4 (66.6%)
Not reported	15 (29.4%)	9 (25.7%)	4 (57.1%)	7 (28.0%)	2 (33.3%)
HRQoL measures					
EQ-5D	16 (31.4%)	7 (20.0%)	3 (42.9%)	10 (36.4%)	1 (16.7%)
EQ-VAS	11 (21.6%)	4 (11.4%)	1 (14.3%)	8 (18.2%)	1 (16.7%)
SF-36	11 (23.9%)	10 (28.6%)	2 (28.6%)	4 (18.0%)	–
SF-12	8 (15.7%)	6 (17.1%)	–	4 (18.0%)	1 (16.7%)
SAQ	8 (15.7%)	8 (22.9%)	2 (28.6%)	–	4 (66.6%)
HeartQoL	5 (9.8 %)	3 (8.6%)	–	4 (18.0%)	–
DASI	3 (5.9 %)	2 (5.7%)	1 (14.3%)	1 (4.0%)	–
KCCQ	2 (3.9 %)	2 (5.7%)	–	2 (8.0%)	–
MacNew	2 (3.9 %)	1 (2.9%)	–	1 (4.0%)	–
WHOQOL-BREF	2 (3.9 %)	1 (2.9%)	–	2 (8.0%)	–
NHP	1 (1.9 %)	1 (2.9%)	–	–	–
QLMI	1 (1.9 %)	–	–	1 (4.0%)	–
Outcomes					
Assessed within 1 year	16 (31.4%)	11 (31.4%)	0 (0)	7 (28.0%)	2 (33.3%)
Assessed within 5 years	24 (45.1%)	16 (45.7%)	5 (71.4%)	16 (64.0%)	2 (33.3%)
Assessed beyond 5 years	12 (23.5%)	8 (22.9%)	2 (28.6%)	2 (8.0%)	2 (33.3%)
Number of events					
At least 25 end points	46 (90.2%)	31 (88.6%)	3 (42.9%)	22 (88.0%)	5 (83.3%)
Not reported	4 (7.8%)	4 (11.4%)	3 (42.9%)	2 (8.0%)	1 (16.7%)
Outcomes adjudicated independently	27 (52.9%)	20 (57.1%)	4 (57.1%)	13 (52.0%)	3 (50%)
Covariates					
Adjusted for ≥10 potential confounding factors	27 (52.9%)	21 (60.0%)	4 (57.1%)	9 (36.0%)	2 (33.3%)
Statistically significant relationship observed (lower HRQoL associated with poorer outcome)	48 (94.1%)	33 (94.3%)	4 (57.1%)	22 (88%)	6 (100%)

ACS, acute coronary syndrome; CABG, coronary artery bypass graft; DASI, Duke Activity Status Index; EQ-5D, EuroQol 5-dimension; EQ-VAS, EuroQol visual analogue scale; HRQoL, health-related quality of life; IHD, ischaemic heart disease; KCCQ, The Kansas City Cardiomyopathy Questionnaire; MACCE, major adverse cardiovascular and cerebrovascular events; MacNew, MacNew Questionnaire; MCS, mental component summary score; MI, myocardial infarction; NHP, Nottingham Health Profile; PCI, percutaneous coronary intervention; PCS, physical component summary score; QLMI, Quality of Life after Myocardial Infarction; SAQ, Seattle Angina Questionnaire; SF-12, 12-Item Short Form Survey; SF-36, 36-Item Short Form Survey; WHOQOL-BREF, WHO Quality of Life Questionnaire, brief version.

Overall, 11 (21.6%) and 25 (49.0%) and 11 (21.6%) of the included articles were published after 2000, 2010 and 2020, respectively, with the latest included article published in 2023.^26^ We found variations in the design, patient population, HRQoL assessment, follow-up duration and outcome definitions between the included studies (figure 2, table 1, online supplemental table S2a, online supplemental table S2b). The start of patient recruitment period ranged from June 1998 to August 2021.

Median study sample size was 1358 participants (IQR 630–3786; range 88–26 641) with a median follow-up of 2 years (IQR 1.0–4.8, range 1 month–14 years). Typically, study participants were middle-aged (mean age 65±5 years (range 53–81 years)), 74% men (IQR 69–78, range 0%–100%). Of the 12 (23.5%) studies describing race, the majority of participants were white (mean proportion 84%, IQR 78–89). Stable IHD was the predominant inclusion criteria (figure 2). Studies of MI frequently included patients following acute MI. Reporting and proportions of particular IHD characteristics, revascularisation procedures, cardiovascular history and risk factors, interventions and comorbidities varied widely (online supplemental table S2a).

### Assessment of HRQoL

HRQoL assessment measures varied with 30 (58.8%), 15 (29.4%) and 6 (11.8%) of studies using only generic, only cardiac-specific and generic and cardiac-specific questionnaires, respectively. Of the 12 HRQoL questionnaires used in studies, the most common was EuroQol 5-dimension (EQ-5D) (16, 31.4%) followed by 36-Item Short Form Survey (SF-36) and EuroQol visual analogue scale (EQ-VAS) (11, 21.6%), 12-Item Short Form Survey (SF-12) and Seattle Angina Questionnaire (SAQ) (8, 15.7%) with Nottingham Health Profile (NHP) and Quality of Life after Myocardial Infarction (QLMI) used in one study each. Frequently, studies reported physical and psychological HRQoL domains or an overall HRQoL score. The reporting, analysis and interpretation of the same HRQoL questionnaire often varied. For example, in 16 studies EQ-5D was analysed as: score per unit increase,^12 27–29^ problems in a domain versus no problem^13 24 30–32^ sum of scores (number of problems),^14 24 25 30 33^ arbitrary cut-offs and centiles or quartiles,^13 24 34^ area under the curve^35^ and changes in time.^32 36^ In six studies, SF-36 was analysed as: continuous variable per unit(s) change, one study as ‘poor’ and ‘good’ categories of overall HRQoL, two studies as tertiles or quartiles,^37 38^ or each domain,^39–41^ changes over time^40^ or the analysis was not specified.^42^

### Risk of bias across studies

**Figure 3:**
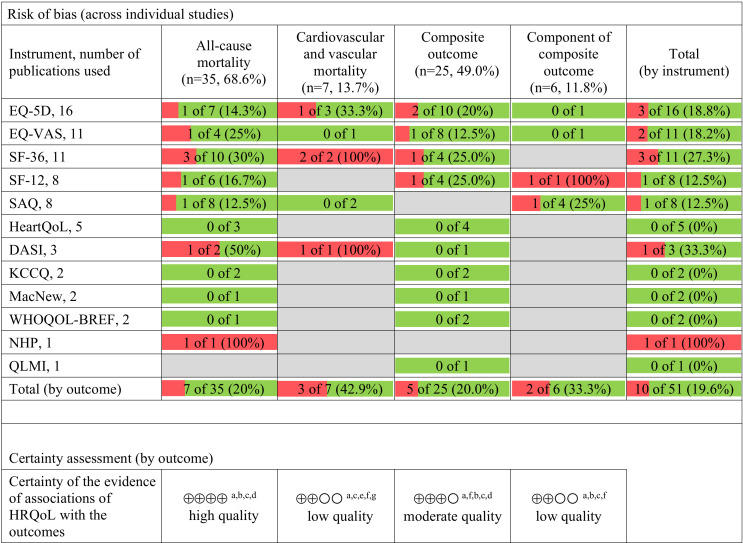
A summary of the number and proportion of publications that were high risk of bias for each HRQoL and outcomes. The assessment of bias was conducted on all the reports that were included in the study. The table displays the number of publications that were identified as having a high risk of bias (low quality). The colours used in the table signify the proportion of reports that were found to have a high risk of bias (red) and those with a low risk of bias (green). Grey-coloured cells represent situations where no publications were available that examined the relationship between the HRQoL measure and the outcome. Certainty assessment was performed using downgrading and upgrading indicators. Downgrading indicators: (a) limitations in study design and/or execution (serious for each outcome, very serious for cardiovascular and vascular mortality due to significant proportion of studies with high risk of bias due to participants’ selection and outcomes assessment); (e) imprecision (low number of studies with positive results only in half of them); (f) inconsistency of effect (a significant proportion of the studies did not show associations with cardiovascular mortality and a component of MACCE outcome; a high statistical heterogeneity (Higgins I^2^ >50%) in meta-analysis of studies of HRQoL and components of MACCE outcome; a relatively small number of trials and heterogeneity of components of MACCE outcome, limiting our ability to draw conclusions); *publication bias was addressed, but considered insufficient to downgrade the quality of evidence. Upgrading indicators: (b) effect size (considering reports showing HR (or OR >2), (c) dose-response gradient (linear associations between HRQoL have been reported or a gradual increase in the effect size presented for more than two categories of HRQoL), (d) adjustment for confounding factors (the estimate of effect is controlled for age, sex and other factors in the majority of reports). DASI, Duke Activity Status Index; EQ-5D, EuroQol 5-dimension; EQ-VAS, EuroQol visual analogue scale; HRQoL, health-related quality life; KCCQ, The Kansas City Cardiomyopathy Questionnaire; MacNew, MacNew Questionnaire; NHP, Nottingham Health Profile; QLMI, Quality of Life after Myocardial Infarction; SAQ, Seattle Angina Questionnaire; SF-12, 12-Item Short Form Survey; SF-36, 36-Item Short Form Survey; WHOQOL-BREF, WHO Quality of Life Questionnaire, brief version.

A summary of the proportion of studies that had high risk of bias (low quality) for each HRQoL/outcomes is shown in figure 3. Six studies did not provide exact estimates for associations between HRQoL and an outcome or its CI, or reported estimates for a selected number of HRQoL domains. More details of risk of bias analysis are given in online supplemental table S3.

### Evidence mapping

**Figure 4:**
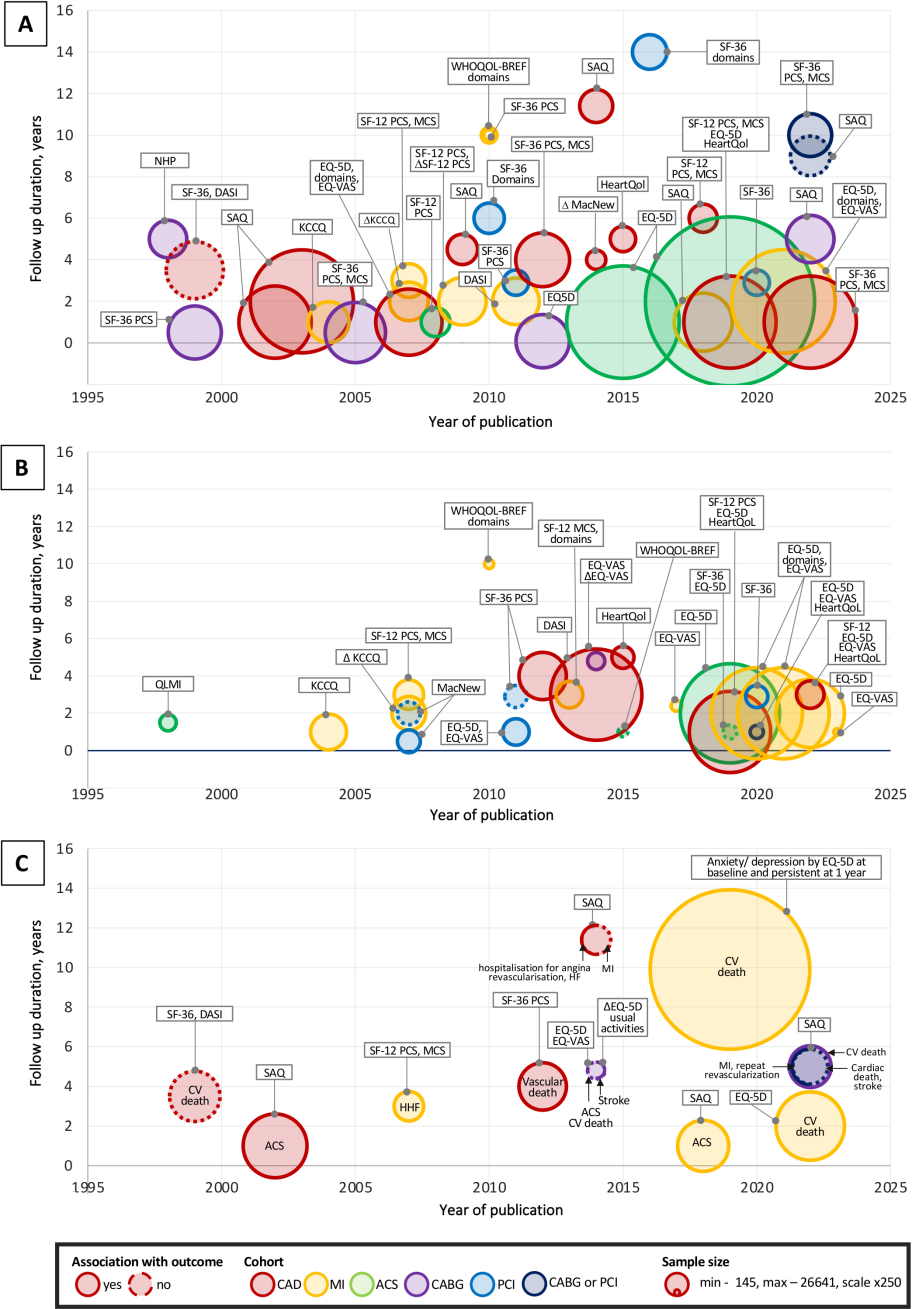
Evidence maps for associations of HRQoL with (A) all-cause mortality, (B) components of MACCE outcomes and (C) CV events for patients with IHD. Associations between HRQoL and outcomes are mapped by publication year of the study results and follow-up duration. Circle colour indicates IHD nosological form, size—sample size, contour—no (dashed) or presence of predictive value of a HRQoL instrument (or domain). If a study reported different results for different HRQoL instruments, associations with outcomes are presented for the instrument with a statistically significantly association. Δ indicates changes in HRQoL score by time. ACS, acute coronary syndrome; CABG, coronary artery bypass graft; CV, cardiovascular; DASI, Duke Activity Status Index; EQ-5D, EuroQol 5-dimension; EQ-VAS, EuroQol visual analogue scale; HHF, hospitalisation for heart failure; HRQoL, health-related quality of life; IHD, ischaemic heart disease; KCCQ, The Kansas City Cardiomyopathy Questionnaire; MacNew, MacNew Questionnaire; MCS, mental component summary score; MI, myocardial infarction; NHP, Nottingham Health Profile; PCI, percutaneous coronary intervention; PCS, physical component summary score; QLMI, Quality of Life after Myocardial Infarction; SAQ, Seattle Angina Questionnaire; SF-12, 12-Item Short Form Survey; SF-36, 36-Item Short Form Survey; WHOQOL-BREF, WHO Quality of Life Questionnaire, brief versio.

Most studies provided effect estimates of the association of HRQoL with survival (n=35, 68.6%) or a composite end point (n=25, 49.0%) (figure 4, online supplemental table S2b). Cardiovascular or vascular mortality was reported in seven (13.7%) studies, and any of stroke, acute coronary syndrome (ACS), MI, heart failure readmission, angina readmission and coronary revascularisation was reported in six studies.

### Descriptive synthesis of included studies

There were 24 (47.1%) studies of associations between the overall HRQoL score and a composite end point (17, 70.8%), all-cause mortality (12, 50%), cardiovascular mortality (2, 8.3%), a component of MACCE—ACS or stroke (1, 4.2%).

#### Association of overall HRQoL score with MACCE

Forty-eight out of 51 (94%) studies reported an inverse association for HRQoL (overall score or a HRQoL domain at baseline or its change by time) and all-cause mortality (high certainty), a composite outcome (moderate certainty), cardiovascular mortality (low certainty) and a cardiovascular event (low certainty) (figure 3).

#### Association of HRQoL domains with MACCE

There were 38 (74.5%) studies reporting prognostic values of HRQoL domains; the majority used SF-36 (11, 28.9%), followed by SF-12 (8, 21.5%), SAQ (8, 21.5%), EQ-5D (7, 18.4%), HeartQoL (3, 5.8%) and WHO-BREF (1, 2.6%), NHP (1, 2.6%) and QLMI (1, 2.6%). Twenty-one (84%) of 25 studies reported associations for both physical and psychological function and the strength or magnitude of the association with MACCE was greater for the physical function.

#### Association of changes in HRQoL with MACCE

There were 6 (11.8%) studies that analysed the predictive value of longitudinal changes of HRQoL; all found an increased risk of MACCE with a decline in HRQoL as measured by generic or cardiac-specific instruments in patients with different IHD states. More results of descriptive synthesis of evidence are reported in the online supplemental material 6.

### Meta-analysis of included studies

**Figure 5:**
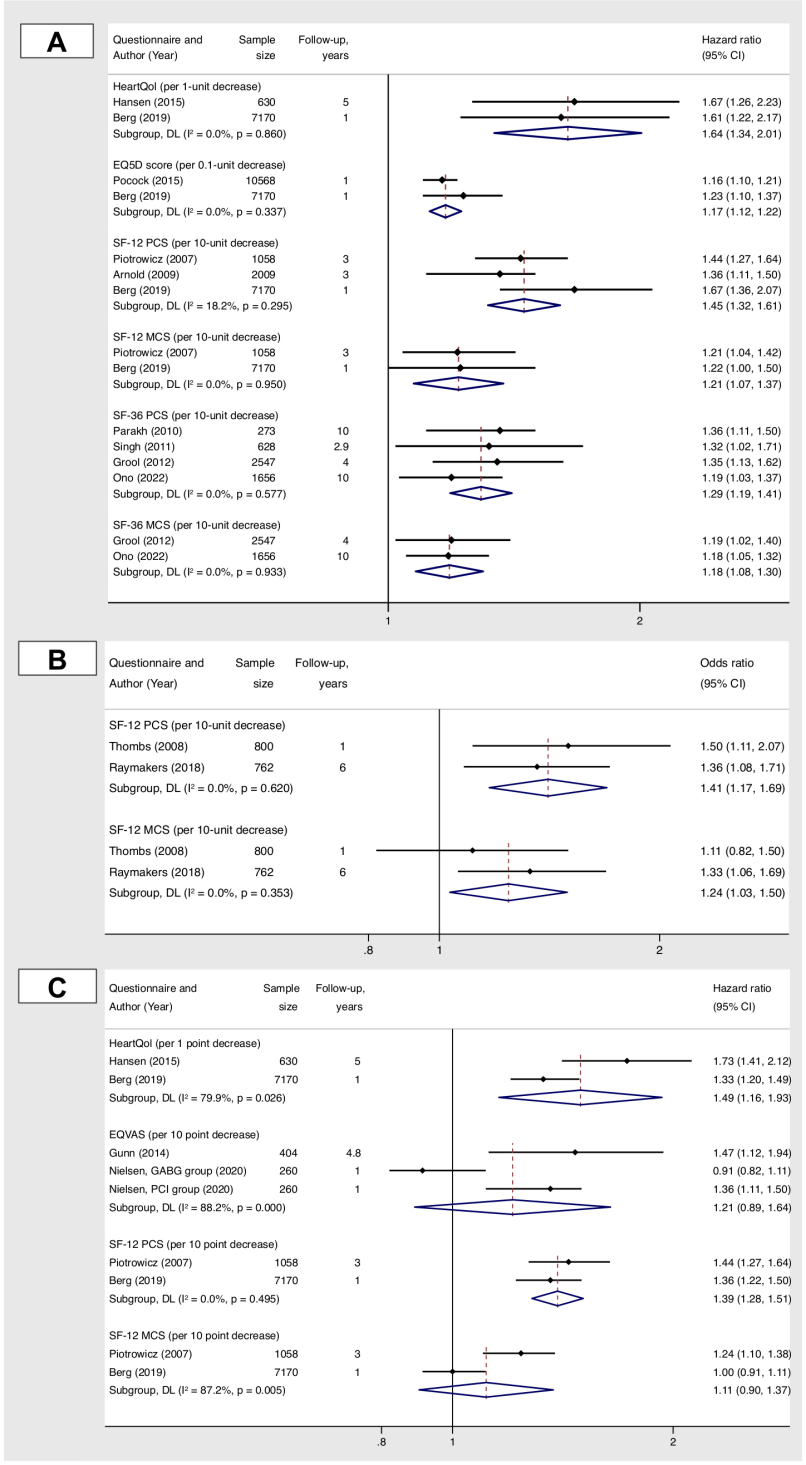
Meta-analysis of studies reporting associations of overall HRQoL scores at baseline with all-cause mortality (panels A and B) and component of MACCE of (panel C). The effect estimates are presented for a difference in baseline HRQoL scores estimated as a continuous variable. Components of MACCE outcome comprised death or cardiac readmissions (MI, HF, stroke, cardiac arrest, ventricular tachycardia or fibrillation, acute CABG)—for HeartQoL; death, stroke or TIA, ACS, acute cardiac readmission or revascularisation—for EQ-VAS; death, hospitalisation due to heart failure, MI, stroke, cardiac arrest, ventricular tachycardia or fibrillation, acute CABG—for SF-12 PCS and SF-12 MCS. ACS, acute coronary syndrome; CABG, coronary artery bypass graft; EQ-5D, EuroQol 5-dimension; EQ-VAS, EuroQol visual analogue scale; HF, heart failure; HRQoL, health-related quality of life; MACCE, major adverse cardiovascular and cerebrovascular events; MCS, mental component summary score; MI, myocardial infarction; PCS, physical component summary score; SF-12, 12-Item Short Form Survey; SF-36, 36-Item Short Form Survey; TIA, transient ischaemic attack.

**Figure 6:**
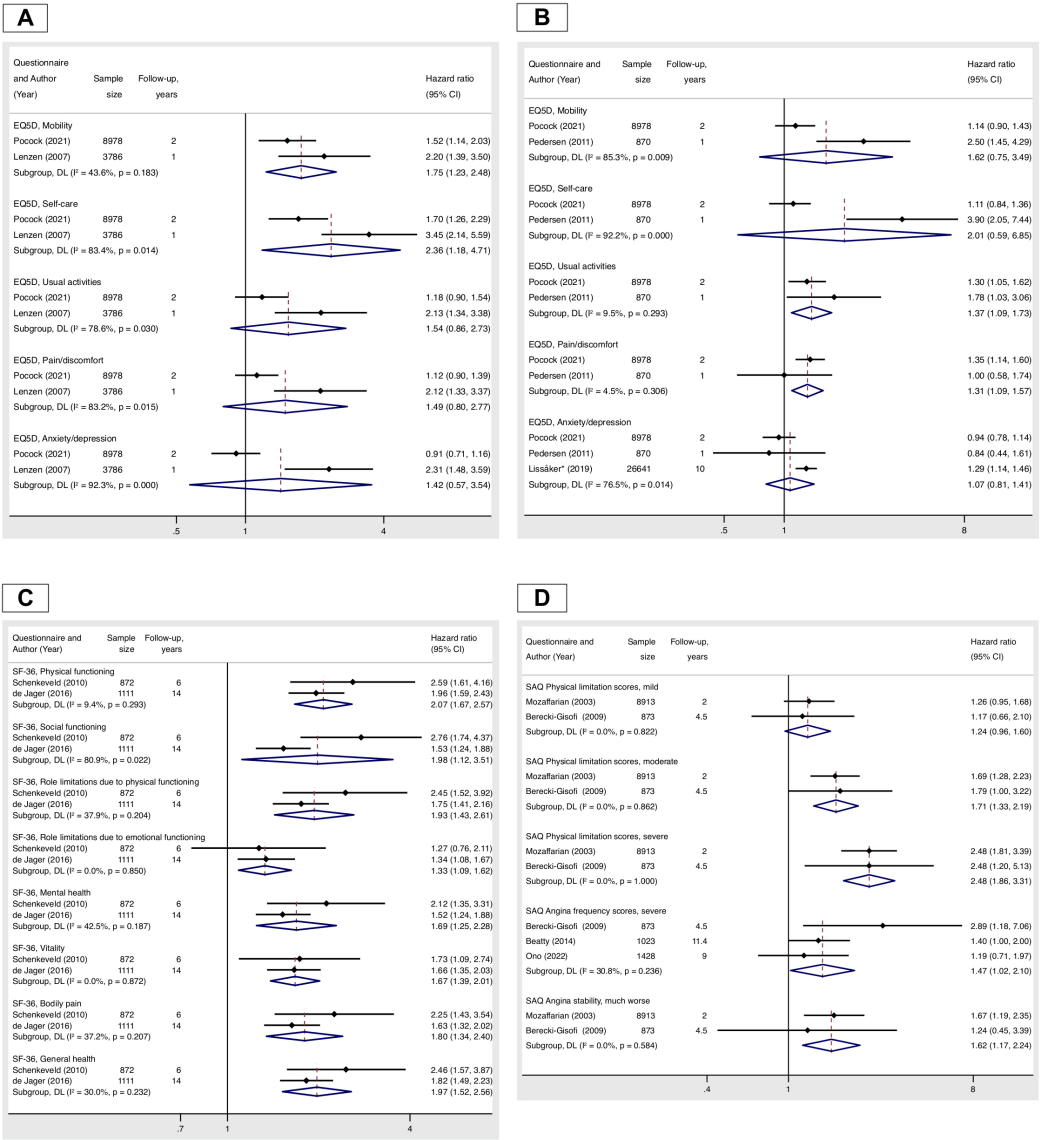
Meta-analysis of studies reporting associations of EQ-5D domains with all-cause mortality (panel A) and components of MACCE outcome of death, myocardial infarction, stroke and unstable angina requiring urgent revascularisation (panel B), SF-36 domains with all-cause mortality (panel C) and SAQ domains with all-cause mortality (panel D). HRs for EQ-5D domains are reported for each domain as categorical variables (‘no problems’ vs ‘moderate or severe problems’), in the study by Lissaker *et al*, the HR for cardiovascular mortality was included. HRs for SF-36 domains are reported for the lowest tertile indicating poor health status versus the other two highest tertiles indicating good health status. HRs for SAQ domains are reported for each domain as categorical variables (indicated level of limitations vs no or minimal limitations (score 75–100)). EQ-5D, EuroQol 5-dimension; MACCE, major adverse cardiovascular and cerebrovascular events; SAQ, Seattle Angina Questionnaire; SF-36, 36-Item Short Form Survey.

There were 23 studies with data amenable for meta-analysis (figures 5–6). A negative impact on overall survival was consistent for poor HRQoL scores, as measured by a range of HRQoL instruments (figure 5). However, there was substantial heterogeneity for associations of HRQoL with composite outcome (figure 5) and for associations of HRQoL domains with an all-cause mortality and a composite outcome (figure 6).

#### Overall HRQoL scores at baseline and all-cause mortality

Pooled effect estimates suggested an increased risk of death with lower baseline HeartQoL score (HR 1.64, 95% CI 1.34 to 2.01); EQ-5D score (HR 1.17, 95% CI 1.12 to 1.22); SF-36 PCS (HR 1.29, 95% CI 1.19 to 1.41); SF-36 MCS (HR 1.18, 95% CI 1.08 to 1.30); SF-12 PCS (HR 1.45, 95% CI 1.32 to 1.61; OR 1.41, 95% CI 1.17 to 1.69) and SF-12 MCS (HR 1.21, 95% CI 1.07 to 1.37; OR 1.24, 95% CI 1.03 to 1.50) (figure 5A and B).

#### Overall HRQoL scores at baseline and composite outcome

Analysis showed a significantly higher risk for composite outcome for patients with lower HeartQoL score (HR 1.49, 95% CI 1.16 to 1.93) and SF-12 PCS (HR 1.39, 95% CI 1.28 to 1.51) (figure 5C).

#### EQ-5D domains and all-cause mortality

Pooled effect estimates for EQ-5D domains showed a higher risk of all-cause mortality with problems with mobility (HR 1.75, 95% CI 1.23 to 2.48) and self-care (HR 2.36, 95% CI 1.18 to 4.71) (figure 6A).

#### EQ-5D domains and a composite outcome or cardiovascular mortality

Pooled effect estimates for EQ-5D domains demonstrated a higher risk of a composite outcomes or cardiovascular mortality for problems with usual activities (HR 1.37, 95% CI 1.09 to 1.73) and pain/discomfort (HR 1.31, 95% CI 1.09 to 1.57) (figure 6B).

#### SF-36 domains and all-cause mortality

Pooled effect estimates for SF-36 domains showed a higher risk of all-cause mortality for each domain, with the highest magnitude of risk for physical functioning (HR 2.07, 95% CI 1.67 to 2.57) (figure 6C).

#### SAQ domains and all-cause mortality

Pooled effect estimates for SAQ domains showed a higher risk of all-cause mortality for moderate (HR 1.71, 95% CI 1.33 to 2.19) and severe physical limitation score (HR 2.48, 95% CI 1.86 to 3.31), severe angina frequency (HR 1.47, 95% CI 1.02 to 2.10) and a significant (much worse) deterioration in angina symptoms (HR 1.62, 95% CI 1.17 to 2.24) (figure 6D).

## Discussion

This systematic review of studies examining HRQoL and outcomes in individuals with IHD found good evidence to support the notion that poor HRQoL as assessed by validated generic or cardiac-specific questionnaires at baseline or its decline over time is associated with an increased risk of MACCE. Of note, however, there was substantial variation in study design, patient population, HRQoL assessment, follow-up duration and outcome definitions, with a focus on all-cause mortality and composite outcomes, and little attention to cardiovascular mortality and non-fatal cardiovascular events.

Poor HRQoL identified even once during the disease course is associated with a higher risk of MACCE which persists over long-term follow-up. Three studies in this review did not identify an inverse association of HRQoL with MACCE. Two of these studies were small, conducted in a predominantly elderly population with ACS and had a low number of events over the follow-up.^42 43^ One of these studies adjusted for frailty status,^42^ and the other did not apply specific inclusion and exclusion criteria^43^; therefore, other factors may have served as more important predictors of events. The third study recruited 2855 patients diagnosed with IHD in 1992–1996 and although it did not find an association of HRQoL with MACCE using validated questionnaires, it reported an inverse association with all-cause and cardiovascular mortality using self-rated health.^41^

We found heterogeneity in the associations of specific HRQoL domains with MACCE. For EQ-5D, ‘mobility’ and ‘self-care’ domains were associated with all-cause mortality, and ‘usual activities’, ‘pain/discomfort’ and ‘anxiety/depression’ were associated with cardiovascular events. The magnitude of the effect on all-cause mortality was the highest for physical functioning as measured by SF-36,^39 40^ with mental health having an effect on cardiovascular mortality. Previous studies also found a higher risk of mortality and MACCE outcomes in patients with a mental health problem, although the association was attenuated by concurrent risk factors.^44 45^ Our findings expand on the results of an earlier systematic review reporting that poor self-rated health was associated with fatal and non-fatal cardiovascular outcomes in individuals with and without prior cardiovascular disease^16^; providing evidence of the prognostic significance of validated HRQoL questionnaires.

Our review suggests that the largest studies used EQ-5D, a questionnaire extensively validated in different populations and simple to complete.^46^ Two multicentre multinational studies of more than 30 000 patients provided compelling evidence for prognostic significance of EQ-5D sum score, problems with some domains (problems with ‘self-care’, ‘mobility’, ‘usual activities’ and ‘pain/discomfort’) and EQ-VAS,^13 27 30 33^ another—established prognostic value of depression patterns in a nationwide cohort of patients with MI in Sweden.^32^ Moreover, both cohorts are the most contemporary and presumably more reflective to patients’ characteristics in current clinical practice.

The mechanisms underlying the association between HRQL and MACCE in patients with IHD remains unknown. Our previous work showed that following MI, HRQoL is lower for older patients, women and in patients with non-ST-elevation myocardial infarction or multimorbidities.^47 48^ Multimorbidity is associated with higher risk of mortality. Previous studies have shown that HRQoL postprocedural in patients with ACS may be affected by complications after procedures, treatment options, revascularisation, socioeconomic status, smoking^49^ and this may impact on patient outcomes. There is also evidence that HRQoL is a mediator of the relationship between medication adherence and hospitalisations and mortality in patients with heart failure.^50^ Wu and Moser^50^ recommended that it is important to assess medication adherence and HRQoL and develop interventions to improve medication adherence and HRQoL. Hurdus *et al*^51^ showed that interventions such as cardiac rehabilitation, physical activity are associated with improved HRQoL, therefore it is important for clinicians to provide these targetted interventions to improve HRQoL and patient outcomes following IHD.

An implication of our findings is that assessment of patient-reported HRQoL might be helpful for more precise health estimation and for the identification of patients with IHD at higher risk of MACCE. Assessment of patient-reported outcomes is emerging as an important target for high-quality patient-centred healthcare.^52^ Patient-reported compared with physician-reported measures have demonstrated higher validity and reproducibility, sensitivity to clinical change and prognostic value.^3 53^

### Strengths and limitations of the study

We adhered to the PRISMA statement and GRADE approach to estimate the certainty of evidence. The qualitative synthesis and stratified meta-analysis were additionally strengthen by evidence mapping. Our study has limitations. First, studies were considerably heterogeneous with differences in study design, particularly targeted population and the instruments and timing used for HRQoL assessment, the adjudication of the study end points and follow-up, limit the validity of combining studies. Moreover, we assumed independence of the effect by time, despite some reports suggested the opposite. Second, generalisability of our finding might be limited due to frequent exclusion of patients with significant comorbidities and severe illness from the studies and predominant participation of patients from high-income countries. Third, despite no language limitations studies in English were only included, so relevant studies published in other languages might be omitted. Finally, current evidence is based on observational data from participants in studies. Whether associations are upheld in non-responders to study invitations, who often have more severe health problems, is unknown. A digital transformation of healthcare with electronic collection of HRQoL across a wider population offers novel opportunities to systematically collect patients’ view about their health. The assessment of HRQoL using a validated instrument has been proposed as a quality indicator for cardiovascular care.^9^

## Conclusion

This systematic review provides the scope of evidence on the associations of patient-reported HRQoL with MACCE in individuals with IHD. Poor baseline HRQoL or HRQoL worsening over time was related to a higher risk of all-cause mortality or a composite outcome, regardless of the HRQoL instrument and the nosological form of IHD studied. These data contribute to a more comprehensive understanding of the value of HRQoL in IHD as a tool allowing to estimate both an individual patient-reported health and risk of future outcomes. An international consensus on and acceptance of a standardised HRQoL assessment in clinical care of individuals with IHD is warranted.

## Data Availability

Data sharing not applicable as no datasets generated and/or analysed for this study. No data are available. All data relevant to the study are included in the article or uploaded as supplementary information.
