# Correction: *Associations of health-related quality of life with major adverse cardiovascular and cerebrovascular events for individuals with ischaemic heart disease: systematic review, meta-analysis and evidence mapping*

**DOI:** 10.1136/openhrt-2023-002452corr1

**Published:** 2023-11-27

Soloveva A, Gale CP, Han NT, *et al*. Associations of health-related quality of life with major adverse cardiovascular and cerebrovascular events for individuals with ischaemic heart disease: systematic review, meta-analysis and evidence mapping. *Open Heart* 2023;10:e002452. doi: 10.1136/openhrt-2023-002452.

This article has been corrected since it was first published. Author name Han Naung Tun has been corrected.

