# Supplementary material for: Associations of health-related quality of life with major adverse cardiovascular and cerebrovascular events for individuals with ischaemic heart disease: systematic review, meta-analysis and evidence mapping
Source: Open Heart. 2023 Oct 27;10(2):e002452. doi: 10.1136/openhrt-2023-002452 (PMC10619110; doi:10.1136/openhrt-2023-002452)
Supplement: Supplementary data [file openhrt-2023-002452supp001.pdf]

Supplementary material to 'Associations of health-related quality of life with major adverse cardiovascular and cerebrovascular events for individuals with ischemic heart disease: Systematic review, meta-analysis and evidence mapping'

SUPPLEMENTARY MATERIAL CONTENT

1. Differences between protocol and review 2
2. Instruments used for the assessment of health-related quality of life 3
3. Electronic search strategy 4
4. Methodology of quality of studies assessment 15
5. Effect estimates transformations 16

Supplementary Table 1. Excluded records among those that had been identified from a previously published systematic review 16

6. Descriptive synthesis of evidence 18

Supplementary Table 2a. Characteristics of included studies and cohorts 20

Supplementary Table 2b. Publications of associations of HRQoL with MACCE included in the systematic review 29

Supplementary Table 3. Risk of bias assessment 47

References 49

Soloveva A, *et al. Open Heart* 2023; 10:e002452. doi: 10.1136/openhrt-2023-002452

Supplementary material to 'Associations of health-related quality of life with major adverse cardiovascular and cerebrovascular events for individuals with ischemic heart disease: Systematic review, meta-analysis and evidence mapping'

1. Differences between protocol and review

Primary published protocol of the systematic review had been updated after the initial literature search was performed. First, provided only few studies published data on HRQoL estimated during the acute stage of MI as well as within post-MI period, we decided to expand the study population and include patients with any type of IHD. Study exposure defined as HRQoL was slightly modified, including some additional generic and cardiac-specific instruments that are frequently used. Also, we initially considered the primary outcome as CVD death, recurring MI, coronary revascularization, stroke, atrial fibrillation, and heart failure. However, on review of the included studies, we made a unanimous decision to change the primary outcome and consider more broader endpoint consisted of all-cause death as well as hospitalization with unstable angina instead of atrial fibrillation (so including more universal 5-component MACCE and death, including cardiovascular death).

We planned to search studies from inception to May 2020, but expanded this period to the most recently published studies. Although we were intended to consider only prospective cohort studies, we included one retrospective study that met the main inclusion criteria.

As for meta-analysis, we also planned to carry out sensitivity analysis to determine whether the overall estimates between HRQoL and MACCE outcomes are influenced by outlier studies, subgroup analysis to determine heterogeneity based on type of HRQoL measure, study follow up period and design. Due to the paucity of available data, we were able to combine only few studies as the instruments used for measuring HRQoL and the scoring algorithms were disparate, as well as statistical approaches to estimate the associations with outcomes.

Soloveva A, *et al. Open Heart* 2023; 10:e002452. doi: 10.1136/openhrt-2023-002452

Supplementary material to 'Associations of health-related quality of life with major adverse cardiovascular and cerebrovascular events for individuals with ischemic heart disease: Systematic review, meta-analysis and evidence mapping'

2. Instruments used for the assessment of health-related quality of life

Generic instruments:

- EuroQol 5-dimension (EQ-5D), EuroQol visual analogue scale (EQ-VAS)
- 36-item Short Form Survey (SF-36)
- 12-item Short Form Survey (SF-12)
- 8-item Short Form Survey (SF-8)
- World Health Organization Quality of Life Questionnaire, brief version (WHOQOL-BREF)
- Nottingham health profile (NHP)
- the Sickness Impact Profile (SIP)

Cardiac / Disease-specific instruments:

- Quality of Life after Myocardial Infarction (QLMI)
- MacNew Questionnaire (QLMI-2, MacNew QLMI)
- Cardiovascular Limitations and Symptoms Profile (CLASP)
- HeartQoL
- Cardiac Quality of Life Index (QLI, quality of life index-cardiac version summary score, Quality of Life Index–Cardiac Version IV)
- Myocardial Infarction Dimensional Assessment Scale (MIDAS)
- Seattle Angina Questionnaire short-form (SAQ-7)
- Duke Activity Status Index (DASI), a simplified (modified) Duke Activity Status Index (M-DASI)
- Minnesota Living with Heart Failure Questionnaire (MLHFQ)
- The Kansas City Cardiomyopathy Questionnaire (KCCQ)
- Chronic Heart Failure Questionnaire (CHFQ)
- the Quality of Life in Severe Heart Failure Questionnaire (QLQ-SHF)

Supplementary material to 'Associations of health-related quality of life with major adverse cardiovascular and cerebrovascular events for individuals with ischemic heart disease: Systematic review, meta-analysis and evidence mapping'

3. Electronic search strategy

Known relevant studies and their included studies were used to identify records within databases. Candidate search terms were identified by looking at words in the titles, abstracts, and subject indexing of those records. A draft search strategy was developed using those terms and additional search terms were identified from the results of that strategy. Search terms were also identified using the PubMed PubReMiner word frequency analysis tool.
A search filter [1 2] was used to identify papers on prognosis and risk assessment papers in Medline and was also used for the other databases. The strategies were peer reviewed by another information specialist prior to execution using the PRESS Checklist[3 4]. Citations identified from the literature searches were imported to EndNote and duplicates were removed.

Final search strategy is presented below:

Sources Searched

We conducted electronic searches for eligible studies within each of the following databases:

- MEDLINE(R) ALL (OvidSP) (1946 to March 31, 2023)
- Embase Classic+Embase (OvidSP) (1947 to 2023 March 31)
- APA PsycINFO (OvidSP) (1806 to March Week 4 2023)
- CINAHL (EBSCO) (1985 to April 3rd, 2023)

Search strategy notes
Search strategy notes: Ovid databases
Search lines ending in a '/' are subject heading searches.
Search lines ending in a '*/' are focused subject heading searches.
Search lines beginning 'exp' are exploded subject heading searches.
Search lines ending in .tw. search in the title and abstract only.
Search lines ending in .ti search in the title
Search lines ending in .ab. are searching in abstract
Search lines ending in .ab./freq=2 are searching in abstract where the term occurs at least twice
or/x-y combines search sets in the range x-y with Boolean operator OR.
* is used for truncation of words.
? is used for an optional wildcard.
y adj/x z searches for where there are up to x words in-between word y and the word z
"xx xx" searches for the exact phrase that is displayed within the quote marks

Database: Ovid MEDLINE(R) ALL <1946 to March 31, 2023>
Search Strategy:
----
1 ((myocardial or heart or post) adj infarct*).ti. (100737)
2 ((myocardial or heart or post) adj infarct*).ab. /freq=2 (53156)
3 ((heart or cardiac or ischemic) adj attack*).ti. (4895)
4 ((heart or cardiac or ischemic) adj attack*).ab. /freq=2 (2594)
5 (cardiovascular adj1 stroke*).ti. (39)
6 (cardiovascular adj1 stroke*).ab. /freq=2 (30)
7 ((acute coronary or stable coronary) adj2 syndrome*).ti. (18365)
8 ((acute coronary or stable coronary) adj2 syndrome*).ab. /freq=2 (5918)
9 heart failure/ (141777)
10 heart failure.ti. (84966)
11 heart failure.ab. /freq=2 (58993)
12 exp myocardial ischemia/ (468291)
13 (myocard* adj5 (ischaemia or ischemia)).ti. (14348)
14 (myocard* adj5 (ischaemia or ischemia)).ab. /freq=2 (10163)
15 angina.ti. (19559)
16 angina.ab. /freq=2 (16929)

Supplementary material to 'Associations of health-related quality of life with major adverse cardiovascular and cerebrovascular events for individuals with ischemic heart disease: Systematic review, meta-analysis and evidence mapping'

17 Coronary Artery Bypass/ (52579)
18 (coronary adj2 (disease or bypass* or "by pass*" or grafting* or thrombo*)).ti. (81430)
19 (coronary adj2 (disease or bypass* or "by pass*" or grafting* or thrombo*)).ab. /freq=2 (46025)
20 (coronary adj1 revasculari?ation).ti. (2331)
21 (coronary adj1 revasculari?ation).ab. /freq=2 (1526)
22 exp Angioplasty/ (64209)
23 angioplast*.ti. (19303)
24 angioplast*.ab. /freq=2 (14437)
25 exp Percutaneous Coronary Intervention/ (64103)
26 (percutaneous coronary adj2 (interven* or revascular*)).ti. (17300)
27 (percutaneous coronary adj2 (interven* or revascular*)).ab. /freq=2 (4521)
28 (pci or ptca).ti. (4437)
29 (pci or ptca).ab. /freq=2 (29030)
30 ((coronary or arterial) adj4 dilat*).ti. (1276)
31 ((coronary or arterial) adj4 dilat*).ab. /freq=2 (912)
32 endoluminal repair*.ti. (91)
33 endoluminal repair*.ab. /freq=2 (52)
34 exp Atherectomy/ (2964)
35 atherectom*.ti. (2073)
36 atherectom*.ab. /freq=2 (1291)
37 or/1-36 [cardiac terms] (724298)
38 "quality of life"/ (262815)
39 "quality of life".tw. (361405)
40 (hqol or h qol or hrqol or hr qol or qol).tw. (70568)
41 (pqol or qls).tw. (453)
42 *self report/ (7577)
43 *health status/ (41592)
44 (("self report*" or " self rat*") adj3 (health or questionnaire* or status)).tw. (46419)
45 ("36-Item Short Form Survey" or "Short Form 36" or "SF 36" or SF36 or "12 Item short form survey" or "Short Form 12" or "SF 12" or "Short Form 6" or "SF 6" or "SF 6D" or "short form health survey" or SF6 or SF36 or WHOQOL or "WHOQOL BREF" or "MacNew Questionnaire" or QLI or "Seattle angina questionnaire" or SAQ or "SAQ 7" or "Duke activity status index" or DASI or "M DASI" or "Nottingham health profile" or NHP or "European Quality of Life 5 Dimensions questionnaire" or "EQ 5D" or EuroQol or "EQ 5D 5L" or " EQ 5D 3L" or "Minnesota Living with Heart Failure Questionnaire" or MLHFQ or "Kansas City Cardiomyopathy Questionnaire" or KCCQ or "Cardiovascular Limitations and Symptoms Profile" or CLASP or QLMI or "Myocardial Infarction Dimensional Assessment Scale" or MIDAS or "MI Dimensional Assessment Scale" or "Chronic Heart Failure Questionnaire" or CHFQ or CHQ or "QLQ-SHF" or "The Sickness Impact Profile" or SIP or HeartQoL).tw. (73824)
46 or/38-45 [quality of life] (529886)
47 validat*.tw. (713112)
48 cohort.tw. (758493)
49 predict*.tw. (1999096)
50 prognos*.tw. (771975)
51 follow up.tw. (1177875)
52 or/47-51 [prognostic factors] (4392281)
53 "major adverse cardiac and cerebrovascular event*".tw. (1419)
54 major CV event*.tw. (241)
55 MACE*.tw. (20807)
56 MACCE*.tw. (2075)
57 cardiac event*.ti. (2251)
58 cardiac event*.ab. /freq=2 (5667)
59 coronary event*.ti. (1252)
60 coronary event*.ab. /freq=2 (1647)
61 cardiovascular disease event*.ti. (266)
62 cardiovascular disease event*.ab. /freq=2 (164)
63 CVD event*.tw. (3625)
64 Mortality/ (49283)
65 mortality.fs. (628229)
66 mortality.tw. (951712)
67 death.ti. (131406)

Supplementary material to 'Associations of health-related quality of life with major adverse cardiovascular and cerebrovascular events for individuals with ischemic heart disease: Systematic review, meta-analysis and evidence mapping'

68 death.ab. /freq=2 (236312)
69 ((hospital or patient*) adj2 readmi*).ti. (2958)
70 ((hospital or patient*) adj2 readmi*).ab. /freq=2 (4292)
71 Patient Readmission/ (22458)
72 (rehospitali?ation* or re-hospitali?ation* or rehospitali?ed or re-hospitali?ed).ti. (1204)
73 (rehospitali?ation* or re-hospitali?ation* or rehospitali?ed or re-hospitali?ed).ab. /freq=2 (3430)
74 (repeat* hospitali?ation* or repeat* hospitali?ed).ti. (92)
75 (repeat* hospitali?ation* or repeat* hospitali?ed).ab. /freq=2 (87)
76 or/53-75 [MACE or MAACE or mortality or repeat hospitalisation] (1609797)
77 37 and 46 and 52 and 76 (3143)

Search Strategy: Embase Classic+Embase <1947 to 2023 March 31>
Search Strategy:

----

1 heart infarction/ (333228)
2 ((myocardial or heart or post) adj infarct*).ti. (140903)
3 ((myocardial or heart or post) adj infarct*).ab. /freq=2 (81757)
4 ((heart or cardiac or ischemic) adj attack*).ti. (6868)
5 ((heart or cardiac or ischemic) adj attack*).ab. /freq=2 (3628)
6 (cardiovascular adj1 stroke*).ti. (62)
7 (cardiovascular adj1 stroke*).ab. /freq=2 (45)
8 acute coronary syndromes/ (72282)
9 ((acute coronary or stable coronary) adj2 syndrome*).ti. (30854)
10 ((acute coronary or stable coronary) adj2 syndrome*).ab. /freq=2 (10091)
11 heart failure/ (311836)
12 heart failure.ti. (140213)
13 heart failure.ab. /freq=2 (105382)
14 exp heart muscle ischemia/ (102304)
15 (myocard* adj5 (ischaemia or ischemia)).ti. (18887)
16 (myocard* adj5 (ischaemia or ischemia)).ab. /freq=2 (14369)
17 exp Angina Pectoris/ (117670)
18 angina.ti. (25001)
19 angina.ab. /freq=2 (25997)
20 coronary artery bypass graft/ (86694)
21 coronary artery bypass surgery/ (18096)
22 coronary artery thrombosis/ (9037)
23 (coronary adj2 (disease or bypass* or "by pass*" or grafting* or thrombo*)).ti. (112580)
24 (coronary adj2 (disease or bypass* or "by pass*" or grafting* or thrombo*)).ab. /freq=2 (69013)
25 heart muscle revascularization/ (38082)
26 (coronary adj1 revasculari?ation).ti. (3264)
27 (coronary adj1 revasculari?ation).ab. /freq=2 (2442)
28 coronary artery thrombosis/ (9037)
29 exp angioplasty/ (102656)
30 angioplast*.ti. (25381)
31 angioplast*.ab. /freq=2 (20221)
32 exp Percutaneous Coronary Intervention/ (125500)
33 (percutaneous coronary adj2 (interven* or revascular*)).tw. (72939)
34 (pci or ptca).ti. (10418)
35 (pci or ptca).ab. /freq=2 (58233)
36 ((coronary or arterial) adj4 dilat*).tw. (9168)
37 endoluminal repair*.ti. (107)
38 endoluminal repair*.ab. /freq=2 (63)
39 Atherectomy/ (4642)
40 atherectom*.ti. (3082)
41 atherectom*.ab. /freq=2 (2068)
42 or/1-41 [cardiac terms] (1162670)
43 "quality of life"/ (609926)
44 "quality of life".tw. (579074)

45 (hqol or h qol or hrqol or hr qol or qol).tw. (128711)
46 (pqol or qls).tw. (742)
47 *self report/ (10196)
48 *health status/ (39042)
49 (("self report*" or " self rat*") adj3 (health or questionnaire* or status)).tw. (58206)
50 ("36-Item Short Form Survey" or "Short Form 36" or "SF 36" or SF36 or "12 Item short form survey" or "Short Form 12" or "SF 12" or "Short Form 6" or "SF 6" or "SF 6D" or "short form health survey" or SF6 or SF36 or WHOQOL or "WHOQOL BREF" or "MacNew Questionnaire" or QLI or "Seattle angina questionnaire" or SAQ or "SAQ 7" or "Duke activity status index" or DASI or "M DASI" or "Nottingham health profile" or NHP or "European Quality of Life 5 Dimensions questionnaire" or "EQ 5D" or EuroQol or "EQ 5D 5L" or " EQ 5D 3L" or "Minnesota Living with Heart Failure Questionnaire" or MLHFQ or "Kansas City Cardiomyopathy Questionnaire" or KCCQ or "Cardiovascular Limitations and Symptoms Profile" or CLASP or QLMI or "Myocardial Infarction Dimensional Assessment Scale" or MIDAS or "MI Dimensional Assessment Scale" or "Chronic Heart Failure Questionnaire" or CHFQ or CHQ or "QLQ-SHF" or "The Sickness Impact Profile" or SIP or HeartQoL).tw. (119373)
51 or/43-50 [quality of life] (890563)
52 prognos*.tw. (1216759)
53 cohort*.tw. (1446475)
54 validat*.tw. (1046883)
55 predict*.tw. (2747497)
56 predict*.tw. (2747497)
57 follow up.tw. (1936770)
58 or/52-57 [prognosis factor terms] (6567754)
59 major adverse cardiac event/ (14738)
60 "major adverse cardiac and cerebrovascular event*".tw. (2336)
61 major CV event*.tw. (573)
62 MACE*.tw. (42425)
63 MACCE*.tw. (4336)
64 cardiac event*.ti. (3776)
65 cardiac event*.ab. /freq=2 (9511)
66 coronary event*.ti. (1713)
67 coronary event*.ab. /freq=2 (2400)
68 "major adverse cardiac and cerebrovascular event*".tw. (2336)
69 mortality.tw. (1475776)
70 death.ti. (163040)
71 death.ab. /freq=2 (362480)
72 ((hospital or patient*) adj2 readmi*).ti. (5007)
73 ((hospital or patient*) adj2 readmi*).ab. /freq=2 (8479)
74 hospital readmission/ (97007)
75 (rehospitali?ation* or re-hospitali?ation* or rehospitali?ed or re-hospitali?ed).ti. (2052)
76 (rehospitali?ation* or re-hospitali?ation* or rehospitali?ed or re-hospitali?ed).ab. /freq=2 (6593)
77 (repeat* hospitali?ation* or repeat* hospitali?ed).ti. (126)
78 (repeat* hospitali?ation* or repeat* hospitali?ed).ab. /freq=2 (161)
79 or/59-78 [mace or macce or mortality or rehospitalisation] (1930028)
80 42 and 51 and 58 and 79 (6555)

Database: APA PsycInfo <1806 to March Week 4 2023>
Search Strategy:
Search Strategy:
----
1 myocardial infarctions/ (3058)
2 ((myocardial or heart or post) adj infarct*).ti. (1697)
3 ((myocardial or heart or post) adj infarct*).ab. /freq=2 (871)
4 ((heart or cardiac or ischemic) adj attack*).ti. (387)
5 ((heart or cardiac or ischemic) adj attack*).ab. /freq=2 (300)
6 (cardiovascular adj1 stroke*).ti. (1)
7 (cardiovascular adj1 stroke*).ab. /freq=2 (0)
8 ((acute coronary or stable coronary) adj2 syndrome*).ti. (405)

9 ((acute coronary or stable coronary) adj2 syndrome*).ab. /freq=2 (122)
10 Cardiovascular Disorders/ (11152)
11 heart disorders/ (10832)
12 heart failure.ti. (2050)
13 heart failure.ab. /freq=2 (1454)
14 ischemia/ (4327)
15 (myocard* adj5 (ischaemia or ischemia)).ti. (126)
16 (myocard* adj5 (ischaemia or ischemia)).ab. /freq=2 (95)
17 Angina Pectoris/ (309)
18 angina.ti. (197)
19 angina.ab. /freq=2 (297)
20 Heart Surgery/ (1611)
21 (coronary adj2 (disease or bypass* or "by pass*" or grafting* or thrombo*)).ti. (2660)
22 (coronary adj2 (disease or bypass* or "by pass*" or grafting* or thrombo*)).ab. /freq=2 (1367)
23 (coronary adj1 revasculari?ation).ti. (18)
24 (coronary adj1 revasculari?ation).ab. /freq=2 (11)
25 angioplast*.ti. (75)
26 angioplast*.ab. /freq=2 (71)
27 (percutaneous coronary adj2 (interven* or revascular*)).ti. (121)
28 (percutaneous coronary adj2 (interven* or revascular*)).ab. /freq=2 (52)
29 (pci or ptca).ti. (41)
30 (pci or ptca).ab. /freq=2 (564)
31 ((coronary or arterial) adj4 dilat*).ti. (8)
32 ((coronary or arterial) adj4 dilat*).ab. /freq=2 (13)
33 endoluminal repair*.ti. (0)
34 endoluminal repair*.ab. /freq=2 (0)
35 atherectom*.ti. (0)
36 atherectom*.ab. /freq=2 (0)
37 or/1-36 [cardiac terms] (30603)
38 "quality of life"/ (46922)
39 "quality of life".tw. (85525)
40 Health Related Quality of Life/ (6839)
41 (hqol or h qol or hrqol or hr qol or qol).tw. (17916)
42 (pqol or qls).tw. (241)
43 *self-report/ (0)
44 *health status/ (1005)
45 ((“self report*" or " self rat*") adj3 (health or questionnaire* or status)).tw. (28579)
46 ("36-Item Short Form Survey" or "Short Form 36" or "SF 36" or SF36 or "12 Item short form survey" or "Short Form 12" or "SF 12" or "Short Form 6" or "SF 6" or "SF 6D" or "short form health survey" or SF6 or SF36 or WHOQOL or "WHOQOL BREF" or "MacNew Questionnaire" or QLI or "Seattle angina questionnaire" or SAQ or "SAQ 7" or "Duke activity status index" or DASI or "M DASI" or "Nottingham health profile" or NHP or "European Quality of Life 5 Dimensions questionnaire" or "EQ 5D" or EuroQol or "EQ 5D 5L" or " EQ 5D 3L" or "Minnesota Living with Heart Failure Questionnaire" or MLHFQ or "Kansas City Cardiomyopathy Questionnaire" or KCCQ or "Cardiovascular Limitations and Symptoms Profile" or CLASP or QLMI or "Myocardial Infarction Dimensional Assessment Scale" or MIDAS or "MI Dimensional Assessment Scale" or "Chronic Heart Failure Questionnaire" or CHFQ or CHQ or "QLQ-SHF" or "The Sickness Impact Profile" or SIP or HeartQoL).tw. (15632)
47 or/38-46 [quality of life] (120605)
48 prognos*.tw. (31397)
49 cohort.tw. (84374)
50 validat*.tw. (123963)
51 predict*.tw. (524836)
52 follow up.tw. (137720)
53 or/48-52 [prognostic factors] (804653)
54 "major adverse cardiac and cerebrovascular event*".tw. (4)
55 major CV event*.tw. (4)
56 MACE*.tw. (958)
57 MACCE*.tw. (5)
58 cardiac event*.ti. (123)
59 cardiac event*.ab. /freq=2 (248)

60 coronary event*.ti. (33)
61 coronary event*.ab. /freq=2 (48)
62 cardiovascular disease event*.ti. (10)
63 cardiovascular disease event*.ab. /freq=2 (3)
64 CVD event*.tw. (165)
65 "Death and Dying"/ (36971)
66 mortality.tw. (48140)
67 death.ti. (18153)
68 death.ab. /freq=2 (29320)
69 ((hospital or patient*) adj2 readmi*).ti. (313)
70 ((hospital or patient*) adj2 readmi*).ab. /freq=2 (407)
71 *hospital admission/ (2488)
72 (rehospitali?ation* or re-hospitali?ation* or rehospitali?ed or re-hospitali?ed).ti. (412)
73 (rehospitali?ation* or re-hospitali?ation* or rehospitali?ed or re-hospitali?ed).ab. /freq=2 (740)
74 (repeat* hospitali?ation* or repeat* hospitali?ed).ti. (14)
75 (repeat* hospitali?ation* or repeat* hospitali?ed).ab. /freq=2 (11)
76 or/54-75 [Major cardiac events or mortality or repeat hospitalisation] (94539)
77 37 and 47 and 53 and 76 (269)

Search Strategy CINAHL EBSCO
Search strategy notes:
Search lines using an MH code are subject heading searches.
Subject heading searches ending in a + are exploded.
Search lines using a TX code search in all text
* is used for truncation of words.
? is used for an optional wildcard.
y Near/z searches for where there are up to x words in-between word y and the word z.
"xx xx" searches for the exact phrase that is displayed within the quote marks

Search Strategy CINAHL, Monday, April 03, 2023 1:20:08 PM

#	Query	Limiters/Expanders	Last Run Via	Results
S48	S21 AND S26 AND S32 AND S47	Search modes - Boolean/Phrase	Interface - EBSCOhost Research Databases<br>Search Screen - Advanced Search<br>Database - CINAHL	2,885
S47	S33 OR S34 OR S35 OR S36 OR S37 OR S38 OR S39 OR S40 OR S41 OR S42 OR S43 OR S44 OR S45 OR S46	Search modes - Boolean/Phrase	Interface - EBSCOhost Research Databases<br>Search Screen - Advanced Search<br>Database - CINAHL	562,604
S46	TX (repeat* hospitali?ation* or repeat* hospitali?ed)	Search modes - Boolean/Phrase	Interface - EBSCOhost Research Databases<br>Search Screen - Advanced Search<br>Database - CINAHL	619
S45	TX (rehospitali?ation* or re-hospitali?ation* or rehospitali?ed or re-hospitali?ed)	Search modes - Boolean/Phrase	Interface - EBSCOhost Research Databases<br>Search Screen - Advanced Search<br>Database - CINAHL	3,979
S44	(MH "Readmission")	Search modes - Boolean/Phrase	Interface - EBSCOhost Research Databases	16,728

Supplementary material to 'Associations of health-related quality of life with major adverse cardiovascular and cerebrovascular events for individuals with ischemic heart disease: Systematic review, meta-analysis and evidence mapping'

			Search Screen - Advanced Search<br>Database - CINAHL	
S43	TX ((hospital or patient*) N2 readmi*)	Search modes - Boolean/Phrase	Interface - EBSCOhost Research Databases<br>Search Screen - Advanced Search<br>Database - CINAHL	9,775
S42	TX (mortality or death)	Search modes - Boolean/Phrase	Interface - EBSCOhost Research Databases<br>Search Screen - Advanced Search<br>Database - CINAHL	539,458
S41	(MH "Mortality")	Search modes - Boolean/Phrase	Interface - EBSCOhost Research Databases<br>Search Screen - Advanced Search<br>Database - CINAHL	Display
S40	TX "CVD event*"	Search modes - Boolean/Phrase	Interface - EBSCOhost Research Databases<br>Search Screen - Advanced Search<br>Database - CINAHL	Display
S39	TX "cardiovascular disease event*"	Search modes - Boolean/Phrase	Interface - EBSCOhost Research Databases<br>Search Screen - Advanced Search<br>Database - CINAHL	Display
S38	TX "coronary event*"	Search modes - Boolean/Phrase	Interface - EBSCOhost Research Databases<br>Search Screen - Advanced Search<br>Database - CINAHL	Display
S37	TX "cardiac event*"	Search modes - Boolean/Phrase	Interface - EBSCOhost Research Databases<br>Search Screen - Advanced Search<br>Database - CINAHL	Display
S36	TX MACCE*	Search modes - Boolean/Phrase	Interface - EBSCOhost Research Databases<br>Search Screen - Advanced Search<br>Database - CINAHL	429
S35	TX MACE	Search modes - Boolean/Phrase	Interface - EBSCOhost Research Databases<br>Search Screen - Advanced Search<br>Database - CINAHL	Display
S34	TX "major CV event"	Search modes - Boolean/Phrase	Interface - EBSCOhost Research Databases<br>Search Screen - Advanced	Display

Supplementary material to 'Associations of health-related quality of life with major adverse cardiovascular and cerebrovascular events for individuals with ischemic heart disease: Systematic review, meta-analysis and evidence mapping'

			Search<br>Database - CINAHL	
S33	(MH "Major Adverse Cardiac Events")	Search modes - Boolean/Phrase	Interface - EBSCOhost Research Databases<br>Search Screen - Advanced Search<br>Database - CINAHL	Display
S32	(S27 OR S28 OR S29 OR S30 OR S31)	Search modes - Boolean/Phrase	Interface - EBSCOhost Research Databases<br>Search Screen - Advanced Search<br>Database - CINAHL	Display
S31	TX "follow up"	Search modes - Boolean/Phrase	Interface - EBSCOhost Research Databases<br>Search Screen - Advanced Search<br>Database - CINAHL	Display
S30	TX predict*	Search modes - Boolean/Phrase	Interface - EBSCOhost Research Databases<br>Search Screen - Advanced Search<br>Database - CINAHL	Display
S29	TX validat*	Search modes - Boolean/Phrase	Interface - EBSCOhost Research Databases<br>Search Screen - Advanced Search<br>Database - CINAHL	Display
S28	TX cohort	Search modes - Boolean/Phrase	Interface - EBSCOhost Research Databases<br>Search Screen - Advanced Search<br>Database - CINAHL	Display
S27	TX prognos*	Search modes - Boolean/Phrase	Interface - EBSCOhost Research Databases<br>Search Screen - Advanced Search<br>Database - CINAHL	Display
S26	S22 or S23 or S24 or S25	Search modes - Boolean/Phrase	Interface - EBSCOhost Research Databases<br>Search Screen - Advanced Search<br>Database - CINAHL	Display
S25	TX (("36-Item Short Form Survey" or "Short Form 36" or "SF 36" or SF36 or "12 Item short form survey" or "Short Form 12" or "SF 12" or "Short Form 6" or "SF 6" or "SF 6D" or "short form health survey" or SF6 or SF36 or WHOQOL or "WHOQOL	Search modes - Boolean/Phrase	Interface - EBSCOhost Research Databases<br>Search Screen - Advanced Search<br>Database - CINAHL	Display

Soloveva A, *et al. Open Heart* 2023; 10:e002452. doi: 10.1136/openhrt-2023-002452

Supplementary material to 'Associations of health-related quality of life with major adverse cardiovascular and cerebrovascular events for individuals with ischemic heart disease: Systematic review, meta-analysis and evidence mapping'

	BREF" or "MacNew Questionnaire" or QLI or "Seattle angina questionnaire" or SAQ or "SAQ 7" or "Duke activity status index" or DASI or "M DASI" or "Nottingham health profile" or NHP or "European Quality of Life 5 Dimensions questionnaire" or "EQ 5D" or EuroQol or "EQ 5D 5L" or " EQ 5D 3L" or "Minnesota Living with Heart Failure Questionnaire" or MLHFQ or "Kansas City Cardiomyopathy Questionnaire" or KCCQ or "Cardiovascular Limitations and Symptoms Profile" or CLASP or QLMI or "Myocardial Infarction Dimensional Assessment Scale" or MIDAS or "MI Dimensional Assessment Scale" or "Chronic Heart Failure Questionnaire" or CHFQ or CHQ or "QLQ-SHF" or "The Sickness Impact Profile" o SIP or HeartQoL)			
S24	TX (hqol or "h qol" or hrqol or hr qol or qol or pqol or qls)	Search modes - Boolean/Phrase	Interface - EBSCOhost Research Databases<br>Search Screen - Advanced Search<br>Database - CINAHL	Display
S23	TX "quality of life" or TX "self report* or TX "self rat*"	Search modes - Boolean/Phrase	Interface - EBSCOhost Research Databases<br>Search Screen - Advanced Search<br>Database - CINAHL	Display
S22	MH "Quality of Life" or MH "Self Report" or MH "Health Status"	Search modes - Boolean/Phrase	Interface - EBSCOhost Research Databases<br>Search Screen - Advanced Search<br>Database - CINAHL	Display
S21	S1 or S2 or S3 or S4 or S5 or S6 or S7 or S8 or S9 or S10 or S11 or S12 or S13 or S14 or S15 or S16 or S17 or S18 or S19 or S20	Search modes - Boolean/Phrase	Interface - EBSCOhost Research Databases<br>Search Screen - Advanced Search<br>Database - CINAHL	Display
S20	(MH "Atherectomy+") or TX atherectom*	Search modes - Boolean/Phrase	Interface - EBSCOhost Research Databases	Display

Supplementary material to 'Associations of health-related quality of life with major adverse cardiovascular and cerebrovascular events for individuals with ischemic heart disease: Systematic review, meta-analysis and evidence mapping'

			Search Screen - Advanced Search<br>Database - CINAHL	
S19	TX endoluminal repair*	Search modes - Boolean/Phrase	Interface - EBSCOhost Research Databases<br>Search Screen - Advanced Search<br>Database - CINAHL	Display
S18	TX ((coronary or arterial) N4 dilat*)	Search modes - Boolean/Phrase	Interface - EBSCOhost Research Databases<br>Search Screen - Advanced Search<br>Database - CINAHL	Display
S17	TX (pci or ptca)	Search modes - Boolean/Phrase	Interface - EBSCOhost Research Databases<br>Search Screen - Advanced Search<br>Database - CINAHL	Display
S16	TX (percutaneous coronary N2 (interven* or revascular*))	Search modes - Boolean/Phrase	Interface - EBSCOhost Research Databases<br>Search Screen - Advanced Search<br>Database - CINAHL	Display
S15	(MH "Percutaneous Coronary Intervention")	Search modes - Boolean/Phrase	Interface - EBSCOhost Research Databases<br>Search Screen - Advanced Search<br>Database - CINAHL	Display
S14	TX angioplast*	Search modes - Boolean/Phrase	Interface - EBSCOhost Research Databases<br>Search Screen - Advanced Search<br>Database - CINAHL	Display
S13	(MH "Angioplasty+")	Search modes - Boolean/Phrase	Interface - EBSCOhost Research Databases<br>Search Screen - Advanced Search<br>Database - CINAHL	Display
S12	TX coronary N1 revasculari?ation	Search modes - Boolean/Phrase	Interface - EBSCOhost Research Databases<br>Search Screen - Advanced Search<br>Database - CINAHL	Display
S11	TX (coronary N2 (disease or bypass* or "by pass*" or grafting* or thrombo*))	Search modes - Boolean/Phrase	Interface - EBSCOhost Research Databases<br>Search Screen - Advanced Search<br>Database - CINAHL	Display
S10	(MH "Coronary Artery Bypass")	Search modes - Boolean/Phrase	Interface - EBSCOhost Research Databases<br>Search Screen - Advanced	Display

Supplementary material to 'Associations of health-related quality of life with major adverse cardiovascular and cerebrovascular events for individuals with ischemic heart disease: Systematic review, meta-analysis and evidence mapping'

			Search<br>Database - CINAHL	
S9	TX angina	Search modes - Boolean/Phrase	Interface - EBSCOhost Research Databases<br>Search Screen - Advanced Search<br>Database - CINAHL	Display
S8	TX (myocard* N5 (ischaemia or ischemia))	Search modes - Boolean/Phrase	Interface - EBSCOhost Research Databases<br>Search Screen - Advanced Search<br>Database - CINAHL	Display
S7	(MH "Myocardial Ischemia+")	Search modes - Boolean/Phrase	Interface - EBSCOhost Research Databases<br>Search Screen - Advanced Search<br>Database - CINAHL	Display
S6	TX heart failure	Search modes - Boolean/Phrase	Interface - EBSCOhost Research Databases<br>Search Screen - Advanced Search<br>Database - CINAHL	Display
S5	MH heart failure	Search modes - Boolean/Phrase	Interface - EBSCOhost Research Databases<br>Search Screen - Advanced Search<br>Database - CINAHL	Display
S4	TX ((acute coronary or stable coronary) N2 syndrome*)	Search modes - Boolean/Phrase	Interface - EBSCOhost Research Databases<br>Search Screen - Advanced Search<br>Database - CINAHL	Display
S3	TX (cardiovascular N1 stroke*)	Search modes - Boolean/Phrase	Interface - EBSCOhost Research Databases<br>Search Screen - Advanced Search<br>Database - CINAHL	Display
S2	TX ((heart or cardiac or ischemic) N1 attack*	Search modes - Boolean/Phrase	Interface - EBSCOhost Research Databases<br>Search Screen - Advanced Search<br>Database - CINAHL	Display
S1	TX (myocardial or heart or post) N1 infarct*)	Search modes - Boolean/Phrase	Interface - EBSCOhost Research Databases<br>Search Screen - Advanced Search<br>Database - CINAHL	

Supplementary material to 'Associations of health-related quality of life with major adverse cardiovascular and cerebrovascular events for individuals with ischemic heart disease: Systematic review, meta-analysis and evidence mapping'

4. Methodology of quality of studies assessment

The quality of the included studies was assessed by The Newcastle - Ottawa Quality Assessment Scale for cohort studies. The scale is based on rating 8 items, categorized into three domains (selection, comparability, outcome). Based on the rating in each domains the studies were considered as good quality (3 or 4 stars in selection domain AND 1 or 2 stars in comparability domain AND 2 or 3 stars in outcome/exposure domain), fair quality (2 stars in selection domain AND 1 or 2 stars in comparability domain AND 2 or 3 stars in outcome/exposure domain) or poor quality (0 or 1 star in selection domain OR 0 stars in comparability domain OR 0 or 1 stars in outcome/exposure domain).

The main issues were high risk of bias due to participants selection 20 [39.2%] studies, including 1 study with an unknown sample size and inclusion of participants older than 65 years old, 19 studies with small sample sizes and/or specific inclusion/exclusion criteria) and outcome assessment 31 [60.8%] studies, including 15 studies not reporting numbers lost to follow-up, 3 studies not reporting number of events of interest, 4 studies not reporting sources for outcomes data, 20 studies performed the assessment via contact with patients or referring physicians, and 3 studies with short follow-up duration of 1 month and 6 months. Based on overall judgement, the majority of studies were of high quality (41, 80.4%) and the rest were of low quality (Table S3).

Soloveva A, *et al. Open Heart* 2023; 10:e002452. doi: 10.1136/openhrt-2023-002452

Supplementary material to 'Associations of health-related quality of life with major adverse cardiovascular and cerebrovascular events for individuals with ischemic heart disease: Systematic review, meta-analysis and evidence mapping'

5. Effect estimates transformations

When the effect estimates reported for different unit increments and(or) its directionality (increase/decrease) we re-calculated hazard ratios (odds ratios) through mathematical transformations of raw estimates derived from each study, such as exponentiation, root extraction, division of one by the estimate (whichever applicable), and log-transformation.

Example 1	Effect estimates
Published value of odds ratio (OR), per 1 point increase	x
Transformation into OR per 1 point decrease	=1/x
Transformation into OR per 10-point decrease	=(1/x)^10
Log-transformation of OR for meta-analysis	=log(x)
Example 2	Effect estimates
Published value of hazard ratio (HR), per 10-point increase	y
Transformation into HR per 10-point decrease	=1/y
Transformation into HR per 1-point decrease	$=\sqrt[10]{(1/y)}$
Log-transformation of HR for meta-analysis	=log(y)

Supplementary Table 1. Excluded records among those that had been identified from a previously published systematic review

**First author and publication year of the studies included in a previously published systematic review** [1]	**Reason for exclusion**	**Explanation**
Westin L, 2005[3]	Other HRQOL instruments used	Self-administered multifaceted quality of life questionnaire was used that was constructed, consisting partly of existing instruments and partly of new sections where no relevant instruments were available. This instrument

Supplementary material to 'Associations of health-related quality of life with major adverse cardiovascular and cerebrovascular events for individuals with ischemic heart disease: Systematic review, meta-analysis and evidence mapping'

		was not validated and was not among the protocol defined HRQoL instruments
Chocron, 2000[5]	Not exclusively IHD population	Patients after open heart surgery were studied, IHD consisted only 60% of the study population
Curtis, 2002[6]	No MACCE outcomes	The study main outcome measures were in-hospital mortality and prolonged length of stay (> 14 days)
Deaton, 1998[7]	No MACCE outcomes	All-cause readmissions were studies, the exact reasons were not indicated
Dixon, 2001[8]	Not exclusively IHD population	High risk cardiac patients were studied, representing a mixed cohort, where IHD constituted 82% of patients
Koch, 2007[9]	Not exclusively IHD population	The study analysed a mixed cohort of patients who underwent isolated CABG with or without valve procedures or isolated valve procedure

Abbreviations: CABG – coronary artery bypass graft, HRQoL – health-related quality of life, IHD – ischemic heart disease, MI – myocardial infarction, HF – heart failure, MACCE – MACCE – major adverse cardiac and cerebrovascular events.

Supplementary material to 'Associations of health-related quality of life with major adverse cardiovascular and cerebrovascular events for individuals with ischemic heart disease: Systematic review, meta-analysis and evidence mapping'

6. Descriptive synthesis of evidence

In the EPICOR and EPICOR Asia studies of 23489 individuals with ACS, a lower baseline EQ-5D score was associated with an increased risk of death [10 11] and a composite of death, non-fatal MI or non-fatal stroke at 2 years[12]. National studies in Denmark and the Netherlands also showed its prognostic value in hospitalized patients with stable IHD [13] and after elective CABG[14], and the TIGRIS study demonstrated an inverse association of EQ-5D and EQ-VAS with all-cause mortality and a composite of cardiovascular death, MI, stroke or unstable angina requiring urgent revascularisation) at 1-3 years after MI[15 16]. In another cohort, EQ-VAS showed prognostic value when used in acute MI[17], angina[18], 1 month after PCI [19] and 6 months after surgery[20]. However, in a multicentre study of 2501 outpatients following CABG surgery the predictive role of EQ-VAS was not confirmed[14].

The HeartQol global score was associated with all-cause mortality and cardiac events in a broad IHD cohort from Denmark at 1year [13 21 22], 3 years[18] and 5 years of follow-up[23]. In 2 post-hoc analyses of the EPHESUS trial[24 25] in out-patients with post-MI heart failure, poor HRQoL as measured by the KCCQ at any of 1, 3 or 6 months, was associated with increased all-cause mortality and the composite endpoint of cardiovascular mortality or heart failure hospitalisation at 1 year[25]. Of 3 studies using DASI score [26-28], 2 found associations between lower functional capacity and higher 2-year mortality following MI[27] and higher risk of death, non-fatal MI or stroke within 3 years of follow-up in stable IHD patients [28]. Small studies of QLMI [29]and its modification MacNew instrument [30] showed associations between lower HRQoL and increased risk of death or cardiac events within 18 and 6 months after MI, respectively.

Supplementary material to 'Associations of health-related quality of life with major adverse cardiovascular and cerebrovascular events for individuals with ischemic heart disease: Systematic review, meta-analysis and evidence mapping'

Association of HRQoL with MACCE by duration of follow-up

Two studies investigated differences in the magnitude of associations between HRQoL and cardiovascular outcomes by follow-up duration[12 30]. Poor HRQoL (lowest tertile of MacNew) post-PCI independently predicted a composite of death, non-fatal MI, CABG surgery and PCI, and a composite of death or non-fatal MI at 6 months, but failed to predict these outcomes at 2 years[30]. Similar associations were found for EQ-5D [12] and KCCQ[10 25] with risk of death at 1 and 2 years after ACS. By contrast, all 12 studies with more than 5 years of follow-up reported an inverse association of HRQoL and MACCE.

Supplementary material to 'Associations of health-related quality of life with major adverse cardiovascular and cerebrovascular events for individuals with ischemic heart disease: Systematic review, meta-analysis and evidence mapping'

Supplementary Table 2a. Characteristics of included studies and cohorts

Study description			Inclusion and exclusion criteria			Clinical and demographic characteristics of participants												
**First author, publication year, study me, recruitment period**	**Country(ies)**	**N**	**Group (time of enrollment after CV event)), disease stage, settings**	**Specific inclusion criteria**	**Specific exclusion criteria**	**Age**	**Male sex, %**	**Race (White)**	**Smoking (current)**	**Htn**	**DM**	**Type of index MI**	**Stable angina**	**Prior MI/ index MI**	**Previous /index PCI**	**Previous / index CABG**	**HF/ reduced or <50% EF**	**TIA/ Stroke**
Lim, 1998, MONICA, Sep 1990 - Dec 1991	Australia	375	ACS (6 months), chronic, outpatient	25-69 years old	no	61	71		29									
Soto, 2004, EPHESUS, Dec 1999-Dec 2001	Argentina, Belgium, Brazil, Canada, France, Germany, the Netherlands, Spain, the United Kingdom, and the United State	1516	MI (between days 3 and 14 after MI), acute, outpatient	EF <40% and either HF (pulmonary rales, venous congestion on chest x-ray, or presence of a third heart sound) or DM	use of potassium-sparing diuretics, a SCr >2.5 mg/dL (220 µmol/L), and a serum K >5.0 mmol/L	64	73,6	91	31,9	55,7	26,7	Q wave 67.5		27,7	/35,7		100	9,6
Piotrowicz, 2007, MADIT-II, Jul 1997 - Nov 2001	US, Germany, Netherlands, Israel	1058	MI (≥1 month), chronic, outpatient	≥ 21 yo, MI ≥1 month before, severe left ventricular dysfunction (EF ≤30%)	ICD indication approved by FDA, NYHA IV; coronary revascularization within the preceding 3 months; MI within the past month, advanced cerebrovascular disease; childbearing age and were not using medically prescribed contraceptive measures; had any condition other than cardiac disease that was associated with a high likelihood of death during the trial; or were unwilling to sign the consent form for participation.	63-65	84,1	86,6	80,8	53,1	34,7		27,2		/44,5	/58,5	100	
Parakh, 2010, NA, Jul 1995 - Dec 1996	US	273	MI, acute, inpatient	no major problems with cognition, no comorbid noncardiac illness likely to cause death within 6 months	not reported	61-66	56,8		28,6	67	34,8	30,4 Q wave 20,9 anterior		30,8	/19.8	/19	/57,9	
Kurdyak, 2011, SESAMI, Dec 1999 - Jun 2002	Canada	1941	MI (30 days), chronic, outpatient	>19 yo and <101, English-speaking Ontario residents, admitted to large-volume Ontario hospitals	no valid Ontario health card number, or transferred into the recruiting site, died early (eg, within 24 hours), severe illness (eg, receiving ventilatory support), language barriers, discharged or transferred early after presentation	62.4	70	84	30	49	24				/7,7	/10,8		
Pedersen, 2007, EXIT, Jul 1996 - Apr 2001	The Netherlands	667	PCI, chronic, outpatient	successful PCI, age 35–68, reporting of exhaustion	severe somatic or mental comorbidity (eg, kidney insufficiency, a 3-year or longer history of major depression); somatization disorder, fibromyalgia or chronic fatigue; participation in other behavioral rehabilitation program;	53	78,3		20,1		11,8		22,6	18,3/			1,6	

Supplementary material to 'Associations of health-related quality of life with major adverse cardiovascular and cerebrovascular events for individuals with ischemic heart disease: Systematic review, meta-analysis and evidence mapping'

Study description			Inclusion and exclusion criteria			Clinical and demographic characteristics of participants												
**First author, publication year, study me, recruitment period**	**Country(ies)**	**N**	**Group (time of enrollment after CV event)), disease stage, settings**	**Specific inclusion criteria**	**Specific exclusion criteria**	**Age**	**Male sex, %**	**Race (White)**	**Smoking (current)**	**Htn**	**DM**	**Type of index MI**	**Stable angina**	**Prior MI/ index MI**	**Previous /index PCI**	**Previous / index CABG**	**HF/ reduced or <50% EF**	**TIA/ Stroke**
					unsuccessful treatment for a recent depression or panic disorder; inability to speak Dutch													
Kosiborod, 2007, EPHESUS, Dec 1999-Dec 2001	Argentina, Belgium, Brazil, Canada, France, Germany, the Netherlands, Spain, the United Kingdom, and the United State	1358	MI (between days 3 and 14 after MI), acute, outpatient	EF <40% and either HF (pulmory rales, venous congestion on chest x-ray, or presence of a third heart sound) or DM	use of potassium-sparing diuretics, a SCr >2.5 mg/dL (220 μmol/L), and a serum K >5.0 mmol/L	64	73,9	90,9		56,3	26,7		43,2	27,5			100	8,8
Lissåker CT., 2019, SWEDEHEART, 2006 - Dec 2015	Sweden	26641	MI (6-10 weeks), chronic, outpatient	First MI <75 years	no	61,7	73,5		31,3	39,3	13				2,3/	1,6/	1,1/26,1	/3,3
Pocock, 2015, EPICOR, Sep 2010 - Mar 2011	Denmark, Norway , Finland, Germany, Poland, Romania, Slovenia, Turkey, Greece, Italy, France, Spain, Luxembuourg, Belgium, the UK, Netherlands, Argentina, Brazil, Mexico, and Venezuela	10568	ACS (24 hours), acute, inpatient	≥18 yo, survived to discharge	"secondary" ACS, any condition/circumstance considered likely to limit the completion of follow-up, any serious/ severe comorbidities limiting life expectancy to less than 6 months, previous enrolment in clinical trial	61,8	75					STEMI 46,8%			/ 64,6 - index CABG or PCI		/9,7	
Pocock, 2019, EPICOR; EPICOR Asia, Sep 2010 - Mar 2011; June 2011 - May 2014	Denmark, Norway , Finland, Germany, Poland, Romania, Slovenia, Turkey, Greece, Italy, France, Spain, Luxembuourg, Belgium, the UK, Netherlands, Argentina, Brazil, Mexico, and Venezuela; China, Hong Kong, India, Malaysia, Singapore, South Korea, Thailand, and Vietnam	23 489	ACS (24 (Epicor) or 48 (Epicor Asia) hours), acute, inpatient	≥18 yo, survived to discharge	ACS event precipitated by, or a complication of, surgery, trauma, or gastrointestinal bleeding, or post–PCI; ACS occurred during hospitalization for other reasons; a condition or circumstance arose that could significantly limit patient follow-up; participation in a RCT; already included in the EPICOR Asia study; concomitant serious/severe comorbidities, which might have limited short-term life expectancy	60.9	75,7					49,2 STEMI	13,9	13,8/	/67	/ 1,6	9,1	
Norekvål, 2010, NA, 1992-1997	Norway	145	MI (3 months-5 years), chronic, outpatient	women, aged 60-80 years, now living at home	serious illness like cancer or stroke, or being cognitively impaired	72	0		28	37	12	Q wave 44	45	23	PCI/CABG 3		38	

Supplementary material to 'Associations of health-related quality of life with major adverse cardiovascular and cerebrovascular events for individuals with ischemic heart disease: Systematic review, meta-analysis and evidence mapping'

Study description			Inclusion and exclusion criteria			Clinical and demographic characteristics of participants												
First author, publication year, study me, recruitment period	Country(ies)	N	Group (time of enrollment after CV event)), disease stage, settings	Specific inclusion criteria	Specific exclusion criteria	Age	Male sex, %	Race (White)	Smoking (current)	Htn	DM	Type of index MI	Stable angina	Prior MI/ index MI	Previous /index PCI	Previous / index CABG	HF/ reduced or <50% EF	TIA/ Stroke
Singh, 2011, NA, Oct 2005 - Sep 2008	US	628	PCI, chronic, inpatient	≥65 yo, undergoing PCI, alive at discharge	history of stroke with residual neurological deficits, severe Parkinson disease, or severe dementia	74,8	69		28,6	67	34,8			30,1/	36/100	25,5/ 0	15,8	13,8
Rumsfeld, 1999, PSOCS, Sep 1992 - Dec 1996	US	2480	CABG, mixed, inpatient	completion of 6 months questionnaire	no	63	99			59	26		66	/2,7	/0.2	/100	12	17
Lenzen, 2007, EHS-CR, Nov 2001 - Mar 2002	Armenia, Austria, Belgium, Bulgaria, Croatia, Switzerland, Cyprus, Czech Republic, Denmark, Germany, Egypt, Spain, Finland, France, United Kingdom, Georgia, Greece, Hungary, Israel, Italy, Lithuania, Luxembourg, The Netherlands, Norway, Poland, Portugal, Romania, Russian Federation, Slovenia, Turkey, Serbia& Montenegro	3786	CAD, mixed, inpatient	>50% diameter stenosis in at least one major epicardial vessel	no	62-69	76		52,9	61,5	23,6	14,6% STEMI	53,8	33,9 /	20,7/ 60	10,8 /20	19,1	7,8
Pedersen, 2011, NA, Feb 2005 - Feb 2006	The Netherlands	870	PCI (1 month), mixed, outpatient	alive 1 month post-PCI	no	62,6	72		24,8	43,6	17			26/33,3	27,9/	8,5/		
Spertus, 2002, the Ambulatory Care Quality Improvement Project, Jan 1997 - Dec 1999	US	4484	CAD, chronic, outpatient	reporting angina, a history of CAD, or a previous coronary event or revascularization procedure were identified as having CAD and were sent the questionnaire	no	67	98,3	85,6	22,3	63,6	28,6		100	52.5%	47,7 + CABG/		19,8	/16,6
Grool, 2012, SMART, Oct 2001 - March 2010	The Netherlands	2547	CAD, chronic, inpatient	history of CABG, or PCI, or MI either previously or at inclusion	age ≥80 years, terminal malignancy, lack of independence in daily activities (Rankin scale >3), lack of fluency in Dutch or referral back to the referring specialist	61	81			66	19							
Thombs, 2008, NA,	Canada	800	ACS, acute, inpatient	no	medically unstable or unable to read or speak English	61,5	66,6		33,9	57,7	25,4			28,5/53,5	9,6	5,3		

Supplementary material to 'Associations of health-related quality of life with major adverse cardiovascular and cerebrovascular events for individuals with ischemic heart disease: Systematic review, meta-analysis and evidence mapping'

Study description			Inclusion and exclusion criteria			Clinical and demographic characteristics of participants												
**First author, publication year, study me, recruitment period**	**Country(ies)**	**N**	**Group (time of enrollment after CV event)), disease stage, settings**	**Specific inclusion criteria**	**Specific exclusion criteria**	**Age**	**Male sex, %**	**Race (White)**	**Smoking (current)**	**Htn**	**DM**	**Type of index MI**	**Stable angina**	**Prior MI/ index MI**	**Previous /index PCI**	**Previous / index CABG**	**HF/ reduced or <50% EF**	**TIA/ Stroke**
Schenkeveld, 2010, the RESEARCH registry, Oct 2001 - Oct 2002	The Netherlands	872	PCI (1 month), mixed, outpatient	survived after PCI	no	62	72		41	32	15		50	36/15	25/100	12/0	11	
Nielsen, 2013, NA, Jan-Dec 2009	Denmark	880	MI (12–14 weeks after discharge), chronic, outpatient	first-time MI	repeated MI (Patients who had been discharged with MI between 1994 and 2008)	67	69,1			30,8	15,2						3,2	3,2/5,6
Gunn, 2014, NA, 2008-2010	Finland	404	CABG, mixed, inpatient + outpatient	elective and urgent or emergent CABG completion of 6 months questionnaire	died within 6 months	67	76,7			97	30			16,1	17.8/	/100	18,8	/3,2
Beatty, 2014, the Heart and Soul, Sep 2000 - Dec 2002	US	1023	CAD, chronic, outpatient	history of MI, revascularization, documented CAD by angiography (evidence of >50% stenosis in >1 coronary vessels) or exercise-induced ischemia by treadmill ECG or stress nuclear perfusion imaging	unable to walk 1 block, ACS within the previous 6 months, or likely moving out of the area within 3 years	65-68	82,1		19,6	70,7	25,9			54%/0	58,8 (any revascularisation)		17,5	
Berecki-Gisolf, 2009, ALSWH, 1996-2004	Australia	873	CAD, chronic, outpatient	older women with self-reported IHD		81	0		32	64	19			40/	33 (any revascularisation)		37	
Hansen, 2015, HeartQoL, Nov-Dec 2005	Denmark	630	CAD, chronic, outpatient	angina or prior MI or ischemic HF	psychiatric disorder and current substance use	65	74,9		24,9	49	16		47,5	51/	45,2/	21,1/	33	7,5
Arnold, 2009, PREMIER, Jan 2003 - June 2004	US	2009	MI, acute, inpatient + outpatient	age ≥18, transfer within the first 24 hours of presentation, survival to hospital discharge	cancer	60-69	36,4	83	65,6	69,7	31,8	STEMI 47,7			/65,4	/13,4	12,7/28,4	7,1
Tang, 2014, GeneBank, 2001 - 2007	US	8987	CAD, chronic, inpatient	undergoing elective diagnostic coronary angiography, no ACS within 30 days of enrollment	known ACS within 30 days of enrollment, unable to comply with or unwilling to follow study protocol	64	68		66	74	38			38/ 0			21/26	/7

Supplementary material to ‘Associations of health-related quality of life with major adverse cardiovascular and cerebrovascular events for individuals with ischemic heart disease: Systematic review, meta-analysis and evidence mapping’

Study description			Inclusion and exclusion criteria			Clinical and demographic characteristics of participants												
**First author, publication year, study me, recruitment period**	**Country(ies)**	**N**	**Group (time of enrollment after CV event)), disease stage, settings**	**Specific inclusion criteria**	**Specific exclusion criteria**	**Age**	**Male sex, %**	**Race (White)**	**Smoking (current)**	**Htn**	**DM**	**Type of index MI**	**Stable angina**	**Prior MI/ index MI**	**Previous /index PCI**	**Previous / index CABG**	**HF/ reduced or <50% EF**	**TIA/ Stroke**
Patel, 2018, TRIUMPH month 1, Apr 2005 - Dec 2008	US	2940	MI (1 month), chronic, outpatient	type 1 acute MI; age ≥18 years; initial presentation/transfer within the first 24 hours of original presentation	cancer	59	66,5	72,5		66	30			20,2/100			7,4	
Bosworth, 1999, MOSS, Jan 1992 - Jan 1996	US	2855	CAD, chronic, inpatient	significant CAD	primary congenital heart disease, primary valvular heart disease, prior percutaneous transluminal angioplasty, repeat catheterization during the same hospital admission, or prior enrollment in the study, English speaking, could not be heart transplant patients, alcoholic patients going through withdrawal, or drug users.	62,5	68	82	77,8	62	7,5							/8
de Jager, 2016, RESEARCH, Sep 2001 - Oct 2002	The Netherlands	1111	PCI, chronic, outpatient	no	no	62,1	73,3		40,6	30,7	14,2		50,4	39,4/11	28,1/	12,5/	/11,9	
Moretti, 2015, STORM, May - July 2012	Italy	156	ACS, acute, inpatient	no	no	71	37		44	76	25			25/	55/	10/		
Ho, 2005, PSOCS, Sep 1992 - Dec 1996	US	exact number not specified	CABG, mixed, inpatient	no	no	65	98,2		12,9	61	29			/2,2			/33,2	
Hofer, 2014, NA, NA	Austria	385	CAD, chronic, inpatient + outpatient	knowledge of the German language, angiographically documented CAD, and the absence of other chronic diseases and severe mental disorder	acute MI during the previous 6 months, documented heart failure	62,8	72,2				13,7							
Compostella, 2017, NA, Jan 2008 - Jun 2012	Italy	184	MI (16 (15-18) days), chronic, outpatient	high-risk STEMI, first MI, treated with primary angioplasty, admitted to the CR unit 16 ± 10 days after a complicated STEMI	echo- or cardiac MRI-documented intracavitary thrombosis, extreme thinning or suspected rupture of the ventricular wall or intra-myocardial bleeding, history of previous MI	60,5-66,5	80,4		38,6	65,2	20,7	100 Stemi high risk		0	7 / 92%	4/	/21,2	
Raymakers, 2018, SPHERE, 2004-2010	Ireland and Northern Ireland	762	CAD, chronic, outpatient	documented MI, CABG or angioplasty, established angi	significant mental or physical illness	67.6	71		21,9	87,5	16,7			49,9				

Supplementary material to 'Associations of health-related quality of life with major adverse cardiovascular and cerebrovascular events for individuals with ischemic heart disease: Systematic review, meta-analysis and evidence mapping'

Study description			Inclusion and exclusion criteria			Clinical and demographic characteristics of participants												
First author, publication year, study me, recruitment period	Country(ies)	N	Group (time of enrollment after CV event)), disease stage, settings	Specific inclusion criteria	Specific exclusion criteria	Age	Male sex, %	Race (White)	Smoking (current)	Htn	DM	Type of index MI	Stable angina	Prior MI/ index MI	Previous /index PCI	Previous / index CABG	HF/ reduced or <50% EF	TIA/ Stroke
Mozaffarian, 2003, ACQUIP, Jan 1997 - Mar 2000	US	8913	CAD, chronic, outpatient	assignement to a primary care provider and at least one clinic visit	no	67,6	98,3	66,4	22,3	63,4	26,7			48,3/	47,9 - any revasc/		17,9	
Ono, 2022, SYNTAXES, Mar 2005 - Apr 2007	Austria, Belgium, Czech Republic, Denmark, Finland, France, Germany, Hungary, Italy, Latvia, Norway, Poland, Portugal, Spain, Sweden, The Netherlands, United Kingdom, USA	1656	PCI and CABG, chronic, inpatient	de novo 3-vessel disease or left-main CAD with ≥50% target vessel stenosis, stable/unstable angina or atypical chest pain, or positive evidence of myocardial ischemia if asymptomatic, eligible for both PCI and CABG	previous PCI or CABG, acute myocardial infarction (MI), or the need for concomitant cardiac surgery	65	77,5		20,4	65,9	25			33,2/	0/51,2	0/48,7		14
Berg, 2019, DenHeart survey, Apr 2013 - Apr 2014	Denmark	7170	CAD, mixed, inpatient	no	< 18 years of age, patients without a Danish civil registration number, who did not understand Danish, unconscious when transferred	65,5	73		15	36	14		56	24/	26/	5/	11	
Batty, 2019, ICON1, Nov 2012 - Dec 2015	UK	280	ACS, acute, inpatient	NSTEACS, aged ≥65 years, undergoing coronary angiography with a view to revascularisation	cardiogenic shock, primary arrhythmia, co-existing significant valvular heart disease, malignancy (with life expectancy ≤1 year), active infection (pneumonia, urinary tract infection, or sepsis of other cause) and inability to provide informed consent, alternative diagnoses after coronary angiography	81 ± 4	60		6,8	73,4	25,3	NSTEMI 100	43,6	60,1/81,1	20,7/	6,8/	8,6	16,8
Herlitz, 1998, NA, Jun 1988 - Jun 1991	Sweden	1290	CABG, mixed, outpatient	isolated CABG	no	64	82		12	36	12		98	60/	4/0	5/100	15	8
Ter Horst, 2012, CORRAD, Jan 2002 - Jun 2011	The Netherlands	2501	CABG, chronic, inpatient	isolated elective CABG	recent MI, urgent surgery	65	79				21,7			/0				
Rosello, 2019, EPICOR, Sep 2010 - Mar 2011	Denmark, Norway , Finland, Germany, Poland, Romania, Slovenia, Turkey, Greece, Italy, France, Spain, Luxembuourg, Belgium, the UK, Netherlands, Argentina, Brazil, Mexico, and Venezuela	10 568	ACS (24 hours), acute, inpatient	≥18 yo, survived to discharge	"secondary" ACS (precipitated by, or occurring as, a complication of surgery, trauma, gastrointestinal bleeding or PCI), or occurring during hospitalization for other reasons), any condition/circumstance considered likely to limit the completion of follow-up, any serious/ severe comorbidities limiting life expectancy to less than 6 months, or previous enrolment in EPICOR, or another clinical trial.	62	75	83,4	36,2	57,9	22,7	STEMI 46,8%	11,7	18,5	14.7 /	5.8/	4,8	4,9

Soloveva A, *et al. Open Heart* 2023; 10:e002452. doi: 10.1136/openhrt-2023-002452

Supplementary material to 'Associations of health-related quality of life with major adverse cardiovascular and cerebrovascular events for individuals with ischemic heart disease: Systematic review, meta-analysis and evidence mapping'

**Study description**			**Inclusion and exclusion criteria**			**Clinical and demographic characteristics of participants**												
**First author, publication year, study me, recruitment period**	**Country(ies)**	**N**	**Group (time of enrollment after CV event)), disease stage, settings**	**Specific inclusion criteria**	**Specific exclusion criteria**	**Age**	**Male sex, %**	**Race (White)**	**Smoking (current)**	**Htn**	**DM**	**Type of index MI**	**Stable angina**	**Prior MI/ index MI**	**Previous /index PCI**	**Previous / index CABG**	**HF/ reduced or <50% EF**	**TIA/ Stroke**
Pocock, 2020, TIGRIS, June 2013 - Nov 2014	Belgium, Denmark, Finland, France, Germany, Italy, Netherlands, Norway, Portugal, Romania, Spain, Turkey, the UK, Australia, China, India, Japan, South Korea, Canada, the United States, Argentina, Brazil, Colombia, Mexico, and Venezuela	8978	MI (1-3 years), chronic, outpatient	≥50 years, stable CAD, a documented history of MI 1–3 years prior to enrolment, at least 1 RF: age ≥65 years, DM med, history of a 2nd prior MI, multivessel CAD and/or non-end-stage CKD	condition or circumstance that could significantly limit the complete follow-up, presence of serious/severe comorbidities that, in the opinion of the investigator, may limit life expectancy to <1 y, current participation in a blinded RCT, patients receiving treatment with ticagrelor beyond 12 months, or off-label	66.9 (8.6)	76		13,9		35,1	STEMI 52,3%	9,96	100/0	/80,7%	/7,4%	11,5	2,1/4,5
Kanwar, 2020 (Cohort 1), 2020, NA, Oct 2005 - Sep 2008	US	629	PCI, chronic, inpatient	≥65 yo, undergoing PCI, alive at discharge	history of stroke with residual neurological deficits, severe Parkinson disease, or severe dementia	74,8	69		28,6	67	34,8			30,1/	36/100	25,5/ 0	15,8	13,8
Nielsen, 2020, DenHeart survey, Apr 2013 - Apr 2014	Denmark	260	CABG and PCI, mixed, inpatient	no	< 18 years of age, patients without a Danish civil registration number, who did not understand Danish, severely ill or unconscious when transferred	66,5	85		8,1	34	16,5			21,5/	18,5/50	around 4/50	8,5/	
Rasmussen, 2022, DenHeart survey, Apr 2013 - Apr 2014	Denmark	7167	CAD, mixed, inpatient	no	< 18 years of age, patients without a Danish civil registration number, who did not understand Danish, unconscious when transferred	65,5	73		15	36	14		56	24/	26/	5/	11	
Pocock, 2021, TIGRIS, June 2013 - Nov 2014	Belgium, Denmark, Finland, France, Germany, Italy, Netherlands, Norway, Portugal, Romania, Spain, Turkey, the UK, Australia, China, India, Japan, South Korea, Canada, the United States, Argentina, Brazil, Colombia, Mexico, and Venezuela	8978	MI (1-3 years), chronic, outpatient	≥50 years, stable CAD, a documented history of MI 1–3 years prior to enrolment, at least 1 RF: age ≥65 years, DM med, history of a 2nd prior MI, multivessel CAD and/or non-end-stage CKD	condition or circumstance that could significantly limit the complete follow-up, presence of serious/severe comorbidities that, in the opinion of the investigator, may limit life expectancy to <1 y, current participation in a blinded RCT, patients receiving treatment with ticagrelor beyond 12 months, or off-label	66.9 (8.6)	76		13,9		35,1	STEMI 52,3%	9,96	100/0	/80,7%	/7,4%	11,5	2,1/4,5
Ono, 2022, SYNTAXES, Mar 2005 - Apr 2007	Austria, Belgium, Czech Republic, Denmark, Finland, France, Germany, Hungary, Italy, Latvia, Norway, Poland, Portugal, Spain, Sweden,	1428	PCI and CABG, chronic, outpatient	de novo 3-vessel disease or left-main CAD with ≥50% target vessel stenosis, stable/unstable angina or atypical chest pain, or positive evidence of myocardial ischemia if	previous PCI or CABG, acute myocardial infarction (MI), or the need for concomitant cardiac surgery	65	78,4		18,6	66,5	23,7		56,9	30,8/0	0/52,4	0/47,6		13,4

Supplementary material to 'Associations of health-related quality of life with major adverse cardiovascular and cerebrovascular events for individuals with ischemic heart disease: Systematic review, meta-analysis and evidence mapping'

Study description			Inclusion and exclusion criteria			Clinical and demographic characteristics of participants												
**First author, publication year, study me, recruitment period**	**Country(ies)**	**N**	**Group (time of enrollment after CV event)), disease stage, settings**	**Specific inclusion criteria**	**Specific exclusion criteria**	**Age**	**Male sex, %**	**Race (White)**	**Smoking (current)**	**Htn**	**DM**	**Type of index MI**	**Stable angina**	**Prior MI/ index MI**	**Previous /index PCI**	**Previous / index CABG**	**HF/ reduced or <50% EF**	**TIA/ Stroke**
	The Netherlands, United Kingdom, USA			asymptomatic, eligible for both PCI and CABG														
Dalsgaard, 2022, DenHeart survey, Apr 2013 - Apr 2014	Denmark	931	CAD, mixed, inpatient	no obstructive CAD	patients who did not understand Danish and patients without a Danish civil registration number. Patients were also excluded for ethical reasons if they were too ill to participate or unconscious upon transfer to another department	68	0		12	39	12			6/			5	
Bishawi, 2022, ROOBY-FS, 2002-2007	USA	2008	CABG, chronic, inpatient	no	clinically significant valve disease (i.e., moderate, moderate- to-severe, or severe valve disease), a status requiring immediate surgery, small target vessels (<1.1 mm in internal diameter) or diffuse coronary disease, clinical reservations of the surgical team regarding patients with risk-factor profiles that predisposed them to an extremely high risk of an adverse event, or the inability or unwillingness of the patient to provide consent	63	100	92.3	33,8	86,2	43,1					/100	23,3	7,70
Bagai, 2022, TIGRIS, June 2013 - Nov 2014	Belgium, Denmark, Finland, France, Germany, Italy, Netherlands, Norway, Portugal, Romania, Spain, Turkey, the UK, Australia, China, India, Japan, South Korea, Canada, the United States, Argentina, Brazil,Colombia, Mexico, and Venezuela	5132	MI (1-3 years), chronic, outpatient	≥65 years, stable CAD, a documented history of MI 1–3 years prior to enrolment, at least 1 RF: age ≥65 years, DMmed, history of a 2nd prior MI, multivessel CAD and/or non-end-stage CKD	condition or circumstance that could significantly limit the complete follow-up, Presence of serious/severe comorbidities that, in the opinion of the investigator, may limit life expectancy to <1 y, Current participation in a blinded RCT, Patients receiving treatment with ticagrelor beyond 12 months, or off-label	72	72.6	74.4	9,7		29,4			100/0			13,1	7,4
Vyshnevska, 2023, NA, Jan 2020 - Aug 2021	Ukraine	88	MI, acute, inpatient	successfully performed primary PCI with epicardial blood flow TIMI III, ≥ 18 years, signed informed consent, and the patient's physical, mental, and territorial possibilities to participate in the trial	decompensated valvular heart disease, active bleeding, hemoglobin level below 90 g/l, severe liver disease, kidney failure (glomerular filtration rate (GFR) < 60 ml/min), mental disorders, drug abuse, alcohol addiction, surgical interventions within two months prior enrollment to the study, planned coronary revascularization three months after the event, myocar- dial infarction in anamnesis, Killip class III-IV, permanent atrial fibrillation, active malignancy	61	75		49	87,5	25	STEMI 100%		/100	/100	/0		

Abbreviations: ACS – acute coronary syndrome, CABG – coronary artery bypass graft, CAD – coronary artery disease, CKD – chronic kidney disease, DM

Supplementary material to 'Associations of health-related quality of life with major adverse cardiovascular and cerebrovascular events for individuals with ischemic heart disease: Systematic review, meta-analysis and evidence mapping'

– diabetes mellitus, IHD – ischemic heart disease, MI – Myocardial Infarction, NSTEMI – Non-ST-Elevation Myocardial Infarction, NSTEACS – Non-ST-Elevation acute coronary syndrome, PCI – percutaneous coronary intervention, RCT – randomised clinical trial, RF – risk factor, STEMI – ST Elevation Myocardial Infarction, EF – left ventricular ejection fraction, HF – heart failure, SCr – serum creatinine, ICD – implantable cardioverter-defibrillator, FDA – Food and Drug Administration, NYHA – New York Heart Association, ECG – electrocardiogram, Htn – arterial hypertension, TIA – transient ischemic stroke, ACQUIP – the Ambulatory Care Quality Improvement Project, ALSWH – ALSWH – Australian Longitudinal Study on Women's Health, CORRAD – Coronary Surgery Database Radboud Hospital, UMCN – the Radboud University Nijmegen Medical Centre, EHS-CR – Euro Heart Survey on Coronary Revascularization, EPHESUS – the Eplerenone Post-AMI Heart Failure Efficacy and Survival Study, EPICOR – long-tErm follow uP of antithrombotic management patterns In acute CORonary syndrome patients, EXIT – the randomized EXhaustion Intervention Trial, ICON1 – The study to Improve Clinical Outcomes in high-risk patieNts with acute coronary syndrome, MADIT-II – he Multi-center Autonomic Defibrillator Implantation Trial II, MONICA – monitoring trends and determinants in cardiovascular disease project, MOSS – the Mediators of Social Support study, PREMIER – the Prospective Registry Evaluating Outcomes After Myocardial Iinfaction: Events and Recovery, PSOCS – Processes, Structures, and Outcomes of Care in Cardiac Surgery, RESEARCH – the Rapamycin-Eluting Stent Evaluated At Rotterdam Cardiology Hospital registry, SESAMI – the Socio-Economic and Acute Myo- cardial Infarction Study, SMART – The Second Manifestations of ARTerial disease study, SPHERE – the Secondary Prevention of Heart Disease in General Practice, STORM – acute coronary Syndrome in paTients end Of life and Risk assesMent study, SYNTAXES – Synergy Between PCI With Taxus and Cardiac Surgery Extended Survival, TIGRIS – long-Term rIsk clinical manaGement and healthcare Resource utilization of stable coronary artery dISease in post-myocardial infarction patients, TRIUMPH – Translational Research Investigating Underlying Disparities in Acute Myocardial Infarction Patients' Health Status

Soloveva A, *et al. Open Heart* 2023; 10:e002452. doi: 10.1136/openhrt-2023-002452

Supplementary material to 'Associations of health-related quality of life with major adverse cardiovascular and cerebrovascular events for individuals with ischemic heart disease: Systematic review, meta-analysis and evidence mapping'

Supplementary Table 2b. Publications of associations of HRQoL with MACCE included in the systematic review

First author, publication year, study name, recruitment period	N	CAD, enrollment time (after ACS/MI), cohort, settings	HRQoL instrument, assessment time points	HRQoL instrument as a variable in survival analysis	FU, years	Outcome(s)	Associations with outcome(s), Effect estimate (95% confidence interval)	Statistical model, confounders included in the model (their total number)
Lim, 1998, MONICA, Sep 1990 - Dec 1991	375	ACS (6 months), chronic, outpatient	QLMI, 6 months post hospitalisation	Categorical (quartiles)	1,5	Composite (death, hospitalisation with congestive HF or suspected MI)	OR for scores (vs high score):<br><br>Global low– 2.63 (1.19-5.84), moderate – 1.89 (0.84-4.23);<br><br>Emotional low – 1.68 (0.80-3.52), moderate 1.22 (0.57-2.60); Physical low – 1.79 (0.86-3.73), moderate 1.07 (0.50-2.31); Social low – 2.16 (1.05-4.45), moderate 1.08 (0.49-2.35)	Adjusted,<br><br>age, sex, MONICA diagnostic category, smoking status, IHD history, SBP, pulse rate, disease severity, revascularisation, rehospitalisation, intervention (11)
Herlitz, 1998, NA, Jun 1988 - Jun 1991	1290	CABG (NA), mixed, outpatient	NHP, prior surgery (median 110 days before)	Response for NHP part 1	5	ACM	Positive reply for the questions:<br><br>'I feel lonely' – OR 1,78 (1,17-2,71);<br><br>'I have difficulty climbing stairs' OR 1,50 (1,02-5,22)	Adjusted,<br><br>LV EF, current smoker, age, congestive HF, diabetes, renal dysfunction, previous CVD and intermittent claudication (8)
Rumsfeld, 1999, PSOCS, Sep 1992 - Dec 1996	2480	CABG, mixed, inpatient	SF-36, before CABG	Continuous values for PCS and MCS (per 10 point decrease)	0,5	ACM	a 10-point lower PCS OR 1.39 (1.11-1.77);<br>a 10-point lower MCS OR 1.09 (0.92-1.29);<br>PCS OR 1.32 (1.04-1.69) and MCS OR 1.09 (0.92-1.29) in another model when PCS and MCS used separately	Adjusted,<br><br>MI within 7 days, prior heart surgery, LV EF, diuretic use, age, 3-vessel CAD, PCS, Creatinine, smoking history (8)
Bosworth, 1999, MOSS, Jan 1992 - Jan 1996	2855	CAD, chronic, inpatient	SF-36, DASI, during admission	Categorical values (versus excellent and good health) of domains (except for general health perceptions), continuous foк DASI	3,5	ACM;<br><br>CAD-related mortality	CAD-related mortality and ACM: NS for 7 domains of SF-36 separately and DASI	Adjusted,<br><br>self-rated health, age, sex, education, income, married, race, cancer, peptic ulcer, hepatitis, depression, arthritis, back ache, asthma, COPD, DM, renal dyalisis, renal isufficiency, alcohol, abdomynal aneurism, Htn, PAD, stroke, disease severity, ISEL, CES-D, CPE, ever smoke (28)

Soloveva A, *et al. Open Heart* 2023; 10:e002452. doi: 10.1136/openhrt-2023-002452

Supplementary material to 'Associations of health-related quality of life with major adverse cardiovascular and cerebrovascular events for individuals with ischemic heart disease: Systematic review, meta-analysis and evidence mapping'

First author, publication year, study name, recruitment period	N	CAD, enrollment time (after ACS/MI), cohort, settings	HRQoL instrument, assessment time points	HRQoL instrument as a variable in survival analysis	FU, years	Outcome(s)	Associations with outcome(s), Effect estimate (95% confidence interval)	Statistical model, confounders included in the model (their total number)
Spertus, 2002, the Ambulatory Care Quality Improvement Project, Jan 1997 - Dec 1999	4484	CAD, chronic, outpatient	SAQ, not specified (some time after visit and enrollment in a clinic)	Categories of: SAQ physical limitation SAQ angina stability SAQ angina frequency SAQ quality of life	1	ACM; hospitalization for ACS	OR for mild/moderate/severe (vs no) for ACM: physical limitation 1.5 (0.9, 2.7) / 2.0 (1.2, 3.5) / 4.0 (2.4, 7.2); angina frequency 0.8 (0.6, 1.2) / 1.2 (0.8, 1.8) / 1.6 (0.9, 2.5). OR for ACS admission: physical limitation 1.5 (0.8, 2.9) / 2.0 (1.1, 3.8) / 1.8 (0.9, 3.6); angina frequency 1.4 (0.9, 2.0) / 2.0 (1.2, 3.2) / 2.2 (1.2, 4.2).	Adjusted, Hospitalisation for ACS - previous hospitalization (ACS), previous hospitalization (other), previous PTCA/CABG (3); ACM – age (per 10 years increase) CHF, cancer, previous hospitalization, DM, stroke (8)
Mozaffarian, 2003, ACQUIP, Jan 1997 - Mar 2000	8913	CAD, chronic, outpatient	SAQ, baseline	Categorical variables of SAQ (0-24; 25-49; 50-74; 75-100)	2	ACM	HR for physical limitation (vs 75–100): 0-24 - 2.48 (1.81–3.39); 25-49 - 1.69 (1.28–2.23); 50-75 - 1.26 (0.95–1.68); for anginal instability scores (vs 75–100): (p for trend <.01): 0–24 – 1.67 (1.19–2.35)	Adjusted, age, race, education, income, DM, smoking status, CHF, MI, coronary revascularization, Htn, renal disease, chronic pulmonary disease, anginal instability, anginal frequency, treatment satisfaction, and effects on quality of life (16)
Soto, 2004, EPHESUS, Dec 1999-Dec 2001	1516	MI (between days 3 and 14 after MI), acute, outpatient	KCCQ, 1, 3 and 6 months after MI	Continuous (per 10 points); 4 categories on the basis of KCCQ-os scores: 0 to <25; 25 to <50, 50 to <75, 75 to 100	1	Composite (CV mortality or hospitalization for a CV event, including recurrent MI, HF, stroke, or ventricular arrhythmia); ACM	HR for composite EP: KCCQ score at 1 month (vs 75-100): 50-75 – 1.08 ((0.83 - 1.40); 25-50 – 1.37 (1.00 - 1.87); <25 – 2.02 (1.24 - 3.27); KCCQ at 1 month per 10-point decline – 1.097; (1.038 to 1.159). HR for ACM: KCCQ score at 1 month (vs 75-100): 50-75 – 1.29 (0.89 - 1.87); 25-50 – 1.65 (1.10 - 2.50); <25 – 2.04 (1.12 - 3.72); KCCQ at 1 month per 10-point decline – 1.0112 (1.0041 – 1.0189) Similar trends in survival for 3 and 6 months KCCQ score	Adjusted, for both models - age, post-MI LV EF, resting heart rate, beta-blocker use, NYHA class, nonwhite race, and the presence of DM, prior HF or MI, and COPD (10)
Ho, 2005, PSOCS, Sep 1992 - Dec 1996	not specified (3160	CABG, mixed, inpatient	SF-36, 72 hours before surgery	MCS and PCS per 10-point decrease	0,5	ACM	PCS per 10-point decrease: OR 1.70, MCS per 10-point decrease: OR 1.17	Adjusted,

Supplementary material to 'Associations of health-related quality of life with major adverse cardiovascular and cerebrovascular events for individuals with ischemic heart disease: Systematic review, meta-analysis and evidence mapping'

First author, publication year, study name, recruitment period	N	CAD, enrollment time (after ACS/MI), cohort, settings	HRQoL instrument, assessment time points	HRQoL instrument as a variable in survival analysis	FU, years	Outcome(s)	Associations with outcome(s), Effect estimate (95% confidence interval)	Statistical model, confounders included in the model (their total number)
	overall cohort)							sex, prior heart surgery, DM, cerebrovascular disease, PAD, current smoker, Htn, COPD, serum creatinine, NYHA class III or IV, Canadian Cardiovascular Society angina class III or IV, MI within 7 days, surgical priority, IV nitroglycerin, intra-aortic balloon pump, LV EF <45%, left main CAD, three-vessel CAD (18)
Piotrowicz, 2007, MADIT-II, Jul 1997 - Nov 2001	1058	MI (≥1 month), chronic, outpatient	SF-12, 1058 patients at the baseline (enrolment), 627 patients – 12 months	≤ Median cutoff; Continuous (per 10-unit decrease)	3	Composite (death or HF hospitalization); Death; Hospitalization due to HF	HR for HF hospitalization: PCS ≥35 - 1.75 (1.31– 2.35); PCS per 10-unit decrease - 1.42 (1.22– 1.64); MCS ≥53 - 1.36 (1.02– 1.81); MCS per 10-unit decrease - 1.24 (1.09– 1.41). HR for composite (HF hospitalisation or death): PCS ≥35 1.84 (1.44– 2.36), PCS per 10-unit decrease - 1.44 (1.27– 1.64), MCS ≥53 1.41 (1.11– 1.80), MCS per 10-unit decrease - 1.24 (1.10– 1.38). HR for Death: PCS ≥35 - 1.89 (1.34– 2.650), PCS per 10-unit decrease - 1.42 (1.19– 1.69), MCS ≥53 1.39 (1.00– 1.93), MCS per 10-unit decrease - 1.21 (1.04– 1.42).	Adjusted, for both models – age, gender, LV EF, NYHA class, blood urea nitrogen (BUN) level, resting heart rate, and treatment group (7)
Pedersen, 2007, EXIT, Jul 1996 - Apr 2001	667	PCI, chronic, outpatient	MacNew, mean 37 days after PCI	Lowest tertile of MacNEw QoL (indicating poor HRQL)	2	Composite (MACE - death, non-fatal MI, CABG, and PCI); death or non-fatal MI; early (<6	HR for poor QoL for early MACE - 2.20 (1.34–3.61), for late MACE - 0.81 (0.50–1.32). HR for poor QoL for early death/non-fatal MI - 2.65 (1.08–6.52), for late death/non-fatal MI - 0.65 (0.30–1.44).	Adjusted, sex, age, comorbidity, CAD history (defined as MI, PCI, or CABG before the index event), multi-vessel disease, smoking, participation in a behavioral

Supplementary material to 'Associations of health-related quality of life with major adverse cardiovascular and cerebrovascular events for individuals with ischemic heart disease: Systematic review, meta-analysis and evidence mapping'

First author, publication year, study name, recruitment period	N	CAD, enrollment time (after ACS/MI), cohort, settings	HRQoL instrument, assessment time points	HRQoL instrument as a variable in survival analysis	FU, years	Outcome(s)	Associations with outcome(s),<br><br>Effect estimate (95% confidence interval)	Statistical model, confounders included in the model (their total number)
						months) vs late events (>6 months);		intervention, and the use of antidepressant medication (8)
Kosiborod, 2007, EPHESUS, Dec 1999-Dec 2001	1358	MI (between days 3 and 14 after MI), acute, outpatient	KCCQ, 1, 3 and 6 months after MI	KCCQ-os at 1 month (continuous – per 5 point decrease and categorical) Absolute change (5-point) in KCCQ-os between the 1- and 3-month assessments, and between 3 and 6 months changes (10 points)	2	1) ACM<br>2) 1-year CV mortality or hospitalization for a CV event, including recurrent MI, HF, stroke, or ventricular arrhythmia	HR for CV endpoint: KCCQ 1-3 months changes (for each 5-point decrease) - 1.11 (1.05 - 1.17); 1-month KCCQ scores (for each 5-point decrease) - 1.10 (1.05 - 1.16).<br><br>HR for ACM: KCCQ 1-3 months changes (for each 5-point decrease) - 1.09 (1.00 - 1.18); 1-month KCCQ scores (for each 5-point decrease) - 1.01 (0.94 to 1.09).<br><br>Nearly identical results for 3-6 months changes	Adjusted,<br><br>for both models: age, gender, race, prior HF, prior MI, prior angina, Htn, dyslipidemia, DM, prior atrial fibrillation, stroke, chronic lung disease, disease severity at the time of randomization (pulmonary edema, Killip class, LV EF after MI, use of reperfusion therapy), BMI at study baseline (1-month visit), and vital signs (heart rate, systolic and diastolic blood pressures), laboratory values (sodium, glomerular filtration rate), and medications at the 1-month visit (ACE inhibitors or angiotensin receptor blockers, beta-blockers, diuretics, and statins), and baseline KCCQ (26)
Lenzen, 2007, EHS-CR, Nov 2001 - Mar 2002	3786	CAD, mixed, inpatient	EQ-5D, EQ-VAS, before discharge	Categorical by domains: no problems'' was coded 0, whereas ''moderate problems'' and ''severe problems''<br>EQ-VAS and EQ score - by 25% centile	1	ACM	OR for mobility 2.2 (1.39 - 3.5); self-care 3.45 (2.14 - 5.59); usual activities 2.13 (1.34 - 3.38); pain or discomfort 2.12 (1.33 - 3.37); anxiety or depression 2.31 (1.48 - 3.59).<br><br>OR for EQ-VAS (≤60) – 2.41 (1.47 - 3.94).<br><br>OR for no problems on all five dimensions – 0.47 (0.28 - 0.81); OR for problems on all dimensions - 3.85 (2.30 - 6.44).	Adjusted,<br><br>age, DM, PAD, previous MI, history of HF, previous CABG, multivessel disease, left main, LV EF <40%, EuroSCORE, PCI, medical treatment, anti-platelet agents, statins and ACE-inhibitors (15)

Supplementary material to 'Associations of health-related quality of life with major adverse cardiovascular and cerebrovascular events for individuals with ischemic heart disease: Systematic review, meta-analysis and evidence mapping'

First author, publication year, study name, recruitment period	N	CAD, enrollment time (after ACS/MI), cohort, settings	HRQoL instrument, assessment time points	HRQoL instrument as a variable in survival analysis	FU, years	Outcome(s)	Associations with outcome(s), Effect estimate (95% confidence interval)	Statistical model, confounders included in the model (their total number)
Thombs, 2008, NA, NA	800	ACS, acute, inpatient	SF-12, 2-5 days after admission	PCS and MCS scores per 1 point change	1	ACM	OR for PCS - 0.96 (0.93–0.99);<br>OR for MCS 0.99 (0,96–1,02).	Adjusted,<br>age, sex, diagnosis of AMI (vs. UA), previous MI, Killip class >1, BDI Total score (6)
Berecki-Gisolf, 2009, ALSWH, 1996-2004	873	CAD, chronic, outpatient	SAQ, enrollment	Categorical SAQ scores grades	4,5	ACM	HR for mild / moderate/ severe (vs minimal) limitations in physical limitation scores - 1.17 (0.66-2.10) / 1.79 (1.00-3.22) / 2.48 (1.20-5.13).<br>HR for mild / moderate/ severe (vs minimal) limitations in angina frequency scores - 1.00 (0.63-1.61) / 0.74 (0.33-1.64) / 2.89 (1.18-7.06).<br>HR for slightly better, unchanged, slightly worse, much worse (vs much better) in angina stability score – 0.97 (0.50-1.89) / 0.94 (0.62-1.43) / 0.87 (0.44-1.73) / 1.24 (0.45-3.39).	Adjusted,<br>age, pulmonary disease, thyroid disease, kidney disease, DM, smoking status, HF, history of MI (8)
Arnold, 2009, PREMIER, Jan 2003 - June 2004	2009	MI, acute, inpatient + outpatient	SF-12, SAQ, baseline and 1 year	Baseline SF-12 PCS score, SAQ angina frequency score and functional decline defined as 5-piont decrease in PCS or being ''too ill'' to provide a follow-up interview	2	ACM	HR for SF-12 PCS score 0.97 (0.96–0.99), SAQ Angina frequency score 1.00 (0.99–1.01); functional decline at 1 year - 2.64 (1.83–3.80)	Adjusted,<br>sex, marital status, race, education level, difficulty accessing medical care, history of CAD (prior MI, PCI, or CABG), Htn, congestive HF, DM, cerebrovascular disease,<br>smoking status, anemia, depressive symptoms (PHQ-9 ≥10), frequency of angina pectoris (assessed using the SAQ), creatinine level, LV systolic dysfunction, ST-elevation MI, Thrombolysis in Myocardial Infarction risk score, quality of care indicators (19)

Supplementary material to 'Associations of health-related quality of life with major adverse cardiovascular and cerebrovascular events for individuals with ischemic heart disease: Systematic review, meta-analysis and evidence mapping'

First author, publication year, study name, recruitment period	N	CAD, enrollment time (after ACS/MI), cohort, settings	HRQoL instrument, assessment time points	HRQoL instrument as a variable in survival analysis	FU, years	Outcome(s)	Associations with outcome(s), Effect estimate (95% confidence interval)	Statistical model, confounders included in the model (their total number)
Parakh, 2010, NA, Jul 1995 - Dec 1996	273	MI, acute, inpatient	SF-36, during the hospitalization	Quartiles of SF-36 PCS and SF-36 PCS continuous scores (per 1 point and per 1 SD)	10	ACM	HR for SF-36 PCS as a continuous variable – 0.97 (0.96-0.99); increase of 1 SD (12.6 points) in SF-36 PCS scores - 0.72 (0.60-0.87).<br>The results did not change when the alternate version of the model was used or after adjusting for measures of depression, anxiety, and social support – HR 0.97 (0.96-0.99)	Adjusted,<br>age, sex, patient lives alone, history of coronary artery disease before, MI, DM, Q wave MI, Coronary artery bypass graft surgery, PCI, Killip class 1 (10)
Norekvål, 2010, NA, 1992-1997	145	MI (3 months-5 years), chronic, outpatient	WHOQOL-BREF, 3 months to 5 years after MI (mean 29 months)	Continous, per 10 point difference - for 4 domains	10	Composite (MACCE – cardiac death, non-fatal MI, and stroke); ACM	HR for MACCE: physical health domain – 1.17 (0.89-1.55), psychological domain 0.64 (0.43-0.95), social relationships domain - 0.67 (0.50-0.92), environmental domain - 1.77 (1.24-2.53), self-rated health (versus satisfied) – dissatisfied/very dissatisfied 2.44 (0.59-10.12), neither satisfied nor dissatisfied 0.77 (0.28-2.10).<br>HR for ACM: physical health domain 1.13 (0.88-1.46), psychological domain 0.60 (0.40-0.90), social relationships domain 1.37 (0.90-2.09), environmental domain 1.90 (1.30-2.77), self-rated health (vs satisfied/very satisfied (ref) - dissatisfied/very dissatisfied 6.26 (1.63-24.01), neither satisfied nor dissatisfied 2.56 (0.86-7.57).	Adjusted,<br>age and time since MI, cohabitation status, serum creatinine, DM, EF, 4 domains, SRH, positive effects of illness, sense of coherence (10)
Schenkeveld, 2010, the RESEARCH registry, Oct 2001 - Oct 2002	872	PCI (1 month), mixed, outpatient	SF-36, at 1 and 12 months post-PCI	Good and poor QoL, decline by half a standard deviation between SF-36 measurements at 1 and 12 months	6	ACM	HR for physical functioning 2.59 (1.61-4.16), social functioning 2.76 (1.74-4.37), role physical functioning 2.45 (1.52-3.92), role emotional functioning 1.27 (0.76-2.11), mental health 2.12 (1.35-3.31), vitality 1.73 (1.09-2.74), bodily pain 2.25 (1.43-3.54), general health 2.46 (1.57-3.87).	Adjusted,<br>gender, age, stent type, previous MI, previous CABG, previous PCI, recent event, multivessel disease, dyslipidemia, hypertension, family history of CAD, current smoking, former smoking, diabetes mellitus,

Supplementary material to 'Associations of health-related quality of life with major adverse cardiovascular and cerebrovascular events for individuals with ischemic heart disease: Systematic review, meta-analysis and evidence mapping'

First author, publication year, study name, recruitment period	N	CAD, enrollment time (after ACS/MI), cohort, settings	HRQoL instrument, assessment time points	HRQoL instrument as a variable in survival analysis	FU, years	Outcome(s)	Associations with outcome(s), Effect estimate (95% confidence interval)	Statistical model, confounders included in the model (their total number)
							HR for decline in physical functioning 0.74 (0.32-1.72); social functioning 1.33 (0.60-2.95), role physical functioning 0.83 (0.37-1.83), role emotional functioning 1.05 (0.48-2.31), mental health 1.48 (0.50-4.44), vitality 1.44 (0.73-2.84), bodily pain 0.77 (0.47-1.76), general health 1.33 (0.67-2.62).	renal impairment, indication for PCI, and LVEF (17)
Kurdyak, 2011, SESAMI, Dec 1999 - Jun 2002	1941	MI (30 days), chronic, outpatient	DASI, 30 days	Continuous DASI score	2	ACM	HR 0.94 (0.91–0.97); HR 0.86 (0.80–0.92) in a sensitivity analysis	Adjusted,<br><br>depression category, age, sex, income category, DM, hypercholesterolemia, Htn, smoking, GRACE prognostic index, DASI, Non-cardiac co-morbidities, PTCA, CABG, Beta-blockers ACE inhibitors, statins, aspirin, nitrates (17)
Singh, 2011, NA, Oct 2005 - Sep 2008	628	PCI, chronic, inpatient	SF-36, before discharge	SF-36 MCS and PCS (per 10 point decrease)	2,9	ACM;<br><br>Composite (ACM or MI)	Death or MI – no significant association with PCS (10-point decrease).<br><br>HR for death for PCS (10-point decrease) - 1.32 (1.02 - 1.71)	Adjusted,<br><br>MCRS (cardiogenic shock, left main CAD, severe renal disease, urgent or emergent procedure, congestive HF class III or higher, thrombus, multivessel disease, and older age), frailty, Charlson index, and SF-36 physical component scores (4)
Pedersen, 2011, NA, Feb 2005 - Feb 2006	870	PCI (1 month), mixed, outpatient	EQ-5D and EQ-VAS, 1 month after PCI	Problems in 5 EQ-5D domains and EQ VAS ≤60 (25 percetile)	1	Composite (ACM or non-fatal MI)	HR for problems (vs no problems) in the domains: mobility 2.50 (1.45–4.29), self-care HR 3.90 (2.05–7.44), usual activities HR 1.78 (1.03–3.06), pain/discomfort - 1.00 (0.58–1.74), anxiety/depression HR 0.84 (0.44–1.61).<br><br>HR for EQ-VAS ≤60 – 2.76 (1.57–4.83)	Adjusted,<br><br>gender, age, indication for PCI, multi-vessel disease, cardiac history (defined as previous MI, PCI, or CABG), comorbidities (defined as hypertension, hypercholesterolemia, or DM),

Supplementary material to 'Associations of health-related quality of life with major adverse cardiovascular and cerebrovascular events for individuals with ischemic heart disease: Systematic review, meta-analysis and evidence mapping'

First author, publication year, study name, recruitment period	N	CAD, enrollment time (after ACS/MI), cohort, settings	HRQoL instrument, assessment time points	HRQoL instrument as a variable in survival analysis	FU, years	Outcome(s)	Associations with outcome(s),<br>Effect estimate (95% confidence interval)	Statistical model, confounders included in the model (their total number)
								smoking, and PCI or CABG during the follow-up period (8)
Grool, 2012, SMART, Oct 2001 - March 2010	2547	CAD, chronic, inpatient	SF-36, baseline	MCS and PCS per 10-point decrease	4	Composite ((recurrent) vascular events – ischemic stroke, MI, retinal infarction and vascular death);<br>vascular death;<br>ACM	HR for MI or Stroke: PCS per 10 point decrease 1.29 (1.11–1.51), MCS per 10 point decrease 1.12 (0.96–1.29).<br>HR for ACM: PCS per 10 point decrease 1.35 (1.13–1.62), MCS per 10 point decrease 1.19 (1.02–1.40).<br>HR for vascular mortality: PCS per 10 point decrease 1.28 (1.00–1.62), MCS per 10 point decrease 1.20 (0.97–1.49)	Adjusted,<br>age, sex, physical activity, smoking (pack-years), alcohol consumption, BMI, hyperlipidaemia, hypertension, DM, intima–media thickness and coexisting locations of symptomatic atherosclerotic disease (11)
Ter Horst, 2012, CORRAD, Jan 2002 - Jun 2011	2501	CABG, chronic, inpatient	EQ-5D, EQ-VAS, on the day before surgery	Continous variables	0,083	ACM	The association between EQ-5D and EQ-VAS and 30-day mortality is significant for EQ-5D (p=0.048; R2 = 0.012), but not for EQ-VAS (p=0.06; R2 = 0.011).<br>C-index for predicting 30-day mortality for EQ-5D -0.39 (0.30– 0.49), for EQ-VAS - 0.35 (0.26–0.44).	Unadjusted
Nielsen, 2013, NA, Jan-Dec 2009	880	MI (12–14 weeks after discharge), chronic, outpatient	SF-12 MCS, 12–14 weeks after discharge with MI	Continuous for domains MCS<br>Categorical – quartiles of MCS	3	Composite (new MI, HF, stroke/TIA and ACM)	HR for MCS quartiles (vs Q4): Q1 2.26 (1.37 - 3.73), Q2 2.15 (1.38 - 3.35), Q3 1.87 (1.21 - 2.88).<br>HR for SF-12 MCS domains (continuous, per one point lower item score): 'Vitality' item 1.24 (1.09 - 1.42), 'Mental Health 1' 1.19 (1.04 - 1.35); 'Mental Health 2' - 1.00 (0.87 - 1.16); 'Role-Emotional 1' – 1.16 (1.04 - 1.29), 'Role-Emotional 2' 1.11 (0.99 - 1.24), Social functioning 1.03 (0.91-1.16)	Adjusted,<br>age, sex, cohabitation status, education, labour market status, MRC dyspnoea score ≥3, history of stroke, DM or HF, secondary prophylactic medication, smoking status, physical activity, HADS-A/D score ≥8 (13)

Supplementary material to 'Associations of health-related quality of life with major adverse cardiovascular and cerebrovascular events for individuals with ischemic heart disease: Systematic review, meta-analysis and evidence mapping'

First author, publication year, study name, recruitment period	N	CAD, enrollment time (after ACS/MI), cohort, settings	HRQoL instrument, assessment time points	HRQoL instrument as a variable in survival analysis	FU, years	Outcome(s)	Associations with outcome(s), Effect estimate (95% confidence interval)	Statistical model, confounders included in the model (their total number)
Gunn, 2014, NA, 2008-2010	404	CABG, mixed, inpatient + outpatient	EQ-5D, EQ-VAS, before elective surgery or immediately postoperatively and at 6 months	Categorical values for EQ-VAS and each domain at baseline and 6 months, continuous values of VAS at 6 month, negative changes in VAS and domains	4,8	Composite (MACCE - stroke or TIA, ACS and cardiac death); Stroke; ACS; CV death	HR for MACCE: worsening usual activities - 2.589 (1.278–5.245), lower 6 months VAS – 0.962 (0.936–0.989), negative change per unit on VAS - 1.047 (1.007–1.089). HR for stroke: worsening usual activities - 2.731 (1.219–6.119). Associations with ACS and CV death – not significant.	Adjusted, age, gender, history of stroke, preoperative AF, LV EF, length of in- hospital stay, postoperative resternotomy, EQ-5D- scores and postoperative stroke (8)
Beatty, 2014, the Heart and Soul, Sep 2000 - Dec 2002	1023	CAD, chronic, outpatient	SAQ, enrollment	SAQ - daily or weekly (vs. no) reported angina; physical limitation entered as a continuous variable	11,4	ACM; Hospitalization for angina; Revascularization; MI; HF	HR for angina frequency daily or weekly (vs. no): death – 1.4 (1.0-2.0), hospitalization for angina - 2.4 (1.6-3.6), revascularization - 1.7 (1.1, 2.7), MI – 0.9 (0.5, 1.7), HF – 1.3 (0.8, 2.1). HR for physical limitation, per 20-unit decrease (worsening): death – 1.1 (1.0-1.3), hospitalization for angina – 1.2 (1.1-1.4), revascularization - 1.2 (1.0-1.4), MI 1.1 (0.9-1.3), HF 1.2 (1.0-1.4)	Adjusted, age, sex, smoking, hypertension history, HF history, BMI, diastolic blood pressure, beta-blocker use, calcium-channel blocker use, and nitrate use, depressive symptoms, treadmill exercise capacity, LV EF, and inducible ischemia (14)
Tang, 2014, GeneBank, 2001 - 2007	8987	CAD, chronic, inpatient	DASI, enrollment	Quartiles of DASI	3	Composite (death, non-fatal MI, or non-fatal stroke)	HR for 7.5-9.8 METs –1.64 (1.25 - 2.14); for 4.8-7.4 METs – 2.47 (1.91 - 3.21); for 1.0-4.7 METs – 3.97 (3.00 - 5.26).	Adjusted, age, sex, systolic blood pressure, low-density lipoprotein cholesterol, high-density lipoprotein cholesterol, smoking, DM, plus history of HF and PAD (9)
Hofer, 2014, NA, NA	385	CAD, chronic, inpatient + outpatient	MacNew, baseline, 1 and 3 months	Categories: deteriorated by ≥0.5 points, unchanged (−0.49 to +0.49), improved by ≥0.50 in global score from baseline and 1 or 3 months	4	ACM	Changes in Global HRQoL ≥0.50 points at 1 month: improved vs. deteriorated 1.70 (1.09, 2.65); improved vs. unchanged 1.91 (0.83, 4.39);	Adjusted, age, angina severity, hypercholesterolemia, DM, physically inactive (5)

Supplementary material to 'Associations of health-related quality of life with major adverse cardiovascular and cerebrovascular events for individuals with ischemic heart disease: Systematic review, meta-analysis and evidence mapping'

First author, publication year, study name, recruitment period	N	CAD, enrollment time (after ACS/MI), cohort, settings	HRQoL instrument, assessment time points	HRQoL instrument as a variable in survival analysis	FU, years	Outcome(s)	Associations with outcome(s), Effect estimate (95% confidence interval)	Statistical model, confounders included in the model (their total number)
							unchanged vs. deteriorated 1.50 (0.72, 3.12).<br>Changes in Global HRQoL ≥0.50 points in 3 months:<br>Improved vs. deteriorated 2.07 (1.29, 3.32);<br>Improved vs. unchanged 2.62 (1.11, 6.17);<br>Unchanged vs. deteriorated 1.63 (0.78, 5.18).	
Pocock, 2015, EPICOR, Sep 2010 - Mar 2011	10568	ACS (24 hours), acute, inpatient	EQ-5D, discharge	EQ-5D score (per unit)	1	ACM	HR for EQ-5D score (per unit) 1.16, 1.10–1.21	Adjusted,<br>age, LV EF <30 and <40%, serum creatinine, cardiac complication in hospital, blood glucose ≥160 mg/dl, COPD, male gender, NSTE-ACS with meds only, NSTE-ACS with PCI/CABG, hemoglobin <13 g/dl, PAD, on diuretics at discharge (15)
Hansen, 2015, HeartQoL, Nov-Dec 2005	630	CAD, chronic, outpatient	HeartQoL, enrollment	HeartQoL as a continuous variable (for global and physical scores) and as categorical variable (for emotional score)	5	ACM;<br>Composite (cardiac readmission for MI, HF, stroke, cardiac arrest)	HR for cardiac readmission (per 1 point decrease, linear association): global score - 1.73 (1.41–2.12), physical score – 1.63 (1.3–1.96); categorical for emotional score (non-linear association, vs high): moderate – 1.60 (1.12–2.30), low score – 1.38 (0.75–2.53).<br>HR for ACM (per 1 point decrease, linear association): lower global HRQoL – 1.67 (1.26–2.23), physical scores – 1.71 (1.33–2.21); categorical for emotional score (non-linear association, vs high): moderate – 1.98 (1.17–3.33), low – 1.38 (0.75–2.53).	Adjusted,<br>age, sex, cohabitation status, cardiac history, disease severity and Charlson's comorbidity index (5)

Supplementary material to 'Associations of health-related quality of life with major adverse cardiovascular and cerebrovascular events for individuals with ischemic heart disease: Systematic review, meta-analysis and evidence mapping'

First author, publication year, study name, recruitment period	N	CAD, enrollment time (after ACS/MI), cohort, settings	HRQoL instrument, assessment time points	HRQoL instrument as a variable in survival analysis	FU, years	Outcome(s)	Associations with outcome(s),<br><br>Effect estimate (95% confidence interval)	Statistical model, confounders included in the model (their total number)
Moretti, 2015, STORM, May - July 2012	156	ACS, acute, inpatient	WHOQOL-BREF, at hospitalization	Not specified	1	Composite (CV death and rehospitalisation for ACS, acute HF, PCI);<br><br>Non-CV events	Unadjusted analysis: the WHOQOL-BREF test failed to provide prognostic information	Unadjusted
de Jager, 2016, RESEARCH, Sep 2001 - Oct 2002	1111	PCI, chronic, outpatient	SF-36, 6 months post PCI	Good vs poor status by each SF-36 domain	14	ACM	Physical functioning (HR: 1.96; 95% CI: 1.59–2.43)<br>Social functioning (HR: 1.53; 95% CI: 1.24–1.88)<br>Role limitations due to physical functioning (HR: 1.75; 95% CI: 1.41–2.16)<br>Role limitations due to emotional functioning (HR: 1.34; 95%CI: 1.08–1.67)<br>Mental health (HR: 1.52; 95% CI: 1.24–1.88)<br>Vitality (HR: 1.66; 95% CI: 1.35–2.03)<br>Bodily pain (HR: 1.63; 95% CI: 1.32–2.02)<br>General health (HR: 1.82; 95% CI: 1.49–2.23)	Adjusted,<br><br>gender, age, dyslipidemia, DM, Htn, family history of CAD, current smoking, former smoking, renal impairment, indication intervention, previous MI, previous PCI, previous CABG, recent event, multivessel disease, LV EF (16)
Compostella, 2017, NA, Jan 2008 - Jun 2012	184	MI (16 (15-18) days), chronic, outpatient	EQ-VAS, during the cardiac rehabilitation period - more than 16 days after MI	Continuous values	2,4	Composite (death or readmission for a new AMI, new revascularization, episodes of HF or stroke)	EQ-VAS was the only psychological variable that maintained a significant correlation with MACE-free survival (p=0.013).	Adjusted,<br><br>age≥65 years, sex, time from event to CR, site of STEMI, signs of HF, number of vessels with critical lesions, incomplete revascularization, NYHA functional class, EF <40 %, known DM, chronic renal failure, physical performance parameters, depression and quality of life parameters (12)

Supplementary material to 'Associations of health-related quality of life with major adverse cardiovascular and cerebrovascular events for individuals with ischemic heart disease: Systematic review, meta-analysis and evidence mapping'

First author, publication year, study name, recruitment period	N	CAD, enrollment time (after ACS/MI), cohort, settings	HRQoL instrument, assessment time points	HRQoL instrument as a variable in survival analysis	FU, years	Outcome(s)	Associations with outcome(s), Effect estimate (95% confidence interval)	Statistical model, confounders included in the model (their total number)
Patel, 2018, TRIUMPH month 1, Apr 2005 - Dec 2008	2940	MI (1 month), chronic, outpatient	SAQ, 1 month	Ranges of the SAQ scores: 0–49 (poor to fair), 50–74 (fair to good) and 75–100 (good to excellent)	1	ACM; ACS rehospitalisations	HR in unadjusted analysis for Men/ Women for ACS hospitalisation: physical limitation (vs Excellent) poor to fair (0 to <50) – 2.4 (1.5, 4.0) / 2.7 (1.6, 4.8), good (50 to <75) 2.1 (1.4, 3.1) / 1.4 (0.7, 2.7); anginal frequency (vs excellent) poor to fair (0 to <50) – 2.5 (1.2, 5.0) / 3.6 (1.8, 7.2), good (50 to <75) 2.3 (1.6, 3.4) / 1.9 (1.2, 3.0); quality of life (vs excellent) – poor to fair (0 to <50) – 2.8 (1.9, 4.1) / 2.1 (1.3, 3.4), good (50 to <75) 1.3 (0.9, 1.8) / 1.4 (0.9, 2.3); SAQ Summary Score (vs excellent) – poor to fair (0 to <50) 2.9 (1.8, 4.8) / 3.1 (1.8, 5.2), good (50 to <75) 2.0 (1.4, 2.9) / 1.8 (1.2, 2.8).<br>HR in unadjusted analysis for Men/ Women for mortality:<br>physical limitation (vs Excellent) poor to fair (0 to <50) – .1 (1.3, 7.0) / 9.1 (3.5, 23.7), good (50 to <75) 21.4 (0.6, 3.5) / 1.2 (0.3, 6.1); anginal frequency (vs excellent) poor to fair (0 to <50) – 21.9 (0.6, 6.1) / 2.4 (0.9, 6.8), good (50 to <75) 1.3 (0.6, 2.6) / 0.9 (0.4, 2.2); quality of life (vs excellent) – poor to fair (0 to <50) – 1.8 (0.9, 3.5) / 1.0 (0.5, 2.3), good (50 to <75) 1.3 (0.7, 2.3) / 1.1 (0.5, 2.2); SAQ Summary Score (vs excellent) – poor to fair (0 to <50) 2.4 (1.1, 5.2) / 2.0 (0.9, 4.6), good (50 to <75) 1.3 (0.7, 2.5) / 1.5 (0.8, 2.9).	Unadjusted
Raymakers, 2018, SPHERE, 2004-2010	762	CAD, chronic, outpatient	SF-12, baseline	PCS and MCS as continuous variables, per 5 point decrease, and as categorical variables (by quintiles)	6	ACM	OR for PCS 0.970 (0.948 - 0.992); for PCS per 5 point decrease 0.86, 0.77–0.96; PCS 1st vs 5th quintiles 3.947 (1.397-11.149).<br>OR for MCS 0.972 (0.949 - 0.994), for MCS per 5 point decrease 0.86 (0.77–	Adjusted,<br>Age, gender, secondary education completed, years since diagnosis, previous MI, DM, diastolic BP > 90 mmHg, 5+ portions of fruit/veg, systolic BP >140 mmHg,

Supplementary material to 'Associations of health-related quality of life with major adverse cardiovascular and cerebrovascular events for individuals with ischemic heart disease: Systematic review, meta-analysis and evidence mapping'

First author, publication year, study name, recruitment period	N	CAD, enrollment time (after ACS/MI), cohort, settings	HRQoL instrument, assessment time points	HRQoL instrument as a variable in survival analysis	FU, years	Outcome(s)	Associations with outcome(s), Effect estimate (95% confidence interval)	Statistical model, confounders included in the model (their total number)
							0.97), MCS 1st vs 5th quintiles 2.961 (1.307-6.709).<br><br>OR for SF-12 domains: physical functioning 0.987 (0.980 -0.994), role—physical functioning 0.988 (0.978 - 0.998), bodily pain 0.995 (0.988 - 1.003), general health 0.977 (0.966 - 0.989), vitality 0.985 (0.976 - 0.993), social functioning 0.989 (0.981 - 0.997), role—emotional functioning 0.990 (0.982 - 0.999), mental health 0.985 (0.974 - 0.995)	cholesterol > 5 mmol/L, current smoker, BMI > 25, Exercise > 15 min/day, study arm, lipid lowering therapy, antihypertensive therapy, anticoagulant therapy, practice ID (18)
Lissåker, 2019, SWEDEHEART, 2006 - Dec 2015	26641	MI (6-10 weeks), chronic, outpatient	EQ-5D, 6-10 weeks and 12-14 months after MI	2 categories of emotional distress (anxiety and depression any at any visit) vs no;<br>3 patterns of emotional distress vs none	9,9	Death (CV and non-CV mortality)	HR for CV mortality for anxiety /depression 6-10 weeks after MI - 1.29 (1.14 - 1.46), for non-CV mortality - 1.34 (1.19 - 1.52).<br><br>HR for CV mortality for patterns of emotional distress (vs none): persistent - 1.45 (1.17 - 1.80), remittent 1.13 (0.90 - 1.41), new - 1.08 (0.82 - 1.43).<br><br>HR for non-CV mortality for patterns of emotional distress (vs none): persistent 1.56 (1.30 - 1.82), remittent 1.00 (0.79 - 1.26), new 1.46 (1.16 - 1.84)	Adjusted,<br><br>age, sex, country of birth, marital status, education, employment, income and smoking, year, DM, Htn, history of HF, history of stroke, previous PCI, previous CABG and hyperlipidaemia, LV EF, cardiac arrest, atrial fibrillation, teaching hospital, heart rate, complications, medications prescribed at discharge (anticoagulants, alpha-2 blockers, angiotensin-converting enzyme inhibitors, other antiplatelets, beta-blockers, calcium antagonists, digitalis, diuretics, statins, other lipid lowering drugs, nitrates) and readmission (34)
Pocock, 2019, EPICOR EPICOR Asia, Epicor Sep 2010 - Mar 2011 Epicor Asia - June 2011 - May 2014	23 489	ACS (24 hours in EPICOR and 48 in EPICOR Asia), acute, inpatient	EQ-5D, discharge	0, 1 and ≥2 problems by EQ-5D	2	ACM	HR for EQ-5D score for the entire cohort: 1 – 1.24 (1.16-1.33), ≥2 – 1.54 (1.35–1.77). In NSTEMI patients: EQ-5D score 1 – 1.19 (1.09-1.30); ≥2 – 1.42 (1.18-1.70). In STEMI patients: EQ-5D score 1	Adjusted,<br><br>age, sex, low LV EF, no coronary revascularization or thrombolysis performed, raised serum creatinine, low haemoglobin, previous cardiac disease, previous COPD or other

Supplementary material to 'Associations of health-related quality of life with major adverse cardiovascular and cerebrovascular events for individuals with ischemic heart disease: Systematic review, meta-analysis and evidence mapping'

First author, publication year, study name, recruitment period	N	CAD, enrollment time (after ACS/MI), cohort, settings	HRQoL instrument, assessment time points	HRQoL instrument as a variable in survival analysis	FU, years	Outcome(s)	Associations with outcome(s), Effect estimate (95% confidence interval)	Statistical model, confounders included in the model (their total number)
							– 1.30 (1.18-1.44); EQ-5D≥2 – 1.69 (1.39-2.06).	chronic lung disease, raised blood glucose, use of either a diuretic or an aldosterone inhibitor at discharge, male sex, low educational level, in-hospital cardiac complications, low BMI, diagnosis of STEMI and Killip class >I (16)
Berg, 2019, DenHeart survey, Apr 2013 - Apr 2014	7170	CAD, mixed, inpatient	SF-12, EQ-5D, HeartQoL, at discharge or 3 days after	SF-12, PCS, per 1 point increase; SF-12, MCS, per 1 point increase; EQ-5D, per 0.1 point increase; HeartQoL Global, per 1 point increase	1	ACM; Composite (CV events – MI, stroke, cardiac arrest, VT/VF, acute CABG)	HR for cardiac events: SF-12 PCS (per 1 point) 0.97 (0.96–0.98), SF-12 MCS (per 1 point) 1.00 (0.99–1.01), EQ-5D (index score per 0.1 point) 0.89 (0.84–0.93), HeartQoL Global (index score per 1 point) 0.75 (0.67–0.83). HR for ACM: SF-12 PCS (per 1 point) 0.95 (0.93–0.97), SF-12 MCS (per 1 point) 0.98 (0.96–1.00), EQ-5D (per 0.1 point) 0.81 (0.73–0.91), HeartQoL Global (per 1 point) 0.62 (0.46–0.82).	Adjusted, age, sex, marital status, educational level, Tu co-morbidity score, smoking, BMI and alcohol intake (8)
Batty, 2019, ICON1, Nov 2012 - Dec 2015	280	ACS, acute, inpatient	SF-36, EQ-5D, at baseline	Not reported	1	Composite (MI, need for urgent repeat revascularisation, stroke, significant bleeding, and ACM)	Not reported for HRQoL Among variables robustly associated with composite end point was problems dressing self - HR 2.96 (1.56 - 5.64)	Adjusted, fried frailty classification, age (categorised as ≥85 years), systolic blood pressure on admission, raised Killip class on admission, history of PAD, problems dressing self, and implantation of a bare metal stent (6)
Rosello, 2019, EPICOR, Sep 2010 - Mar 2011	10 568	ACS (24 hours), acute, inpatient	EQ-5D, discharge	Categories with 0, 1 and ≥2 problems by EQ-5D	2	Composite (death, non-fatal MI, non-fatal stroke)	HR at 1 year: EQ-5D 1 problem – 1.11 (0.86-1.43), EQ-5D ≥2 problems –1.41 (1.14-1.75). HR at 2 years: EQ-5D 1 problem – 0.8 (0.62-1.20); EQ-5D ≥ 2 problems 1.22 (0.93-1.60).	Adjusted, age, sex, education, region, LV EF, BMI, revascularization or thrombolysis, creatinine, Hb, glucose, prior cardiac disease, previous COPD, on diuretics at discharge, on aldosterone inhibitor at discharge, in-hospital cardiac

Supplementary material to 'Associations of health-related quality of life with major adverse cardiovascular and cerebrovascular events for individuals with ischemic heart disease: Systematic review, meta-analysis and evidence mapping'

First author, publication year, study name, recruitment period	N	CAD, enrollment time (after ACS/MI), cohort, settings	HRQoL instrument, assessment time points	HRQoL instrument as a variable in survival analysis	FU, years	Outcome(s)	Associations with outcome(s), Effect estimate (95% confidence interval)	Statistical model, confounders included in the model (their total number)
								complications, STEMI, Killip class (17)
Pocock, 2020, TIGRIS, June 2013 - Nov 2014	8978	MI (1-3 years), chronic, outpatient	EQ-5D, EQ-VAS, enrollment	Categorical EQ-5D overall score (0-10 - from no problems to severe problem in all 5 domains), categorical EQ-VAS (<60, 60-69, 70-79, 80-89, 90-100), dimentions	2	Composite (ACM, MI, stroke and UA requiring urgent revascularisation)	IRR* for EQ-5D overall score (vs 0): 1 – 1.22 (0.97 - 1.53), 2 – 1.46 (1.14 - 1.88), 3 – 1.96 (1.50 - 2.57), 4-10 – 2.86 (2.25 - 3.63).<br>IRR* for problems in EQ-5D domains (for some and severe respectively): mobility 1.87 (1.58 to 2.22) and 5.01 (1.85 to 13.58); self-care 2.14 (1.67 to 2.75) and 3.18 (1.57 to 6.44), usual activities 1.89 (1.57 to 2.26) and 3.34 (2.19 to 5.09), pain 1.71 (1.45 to 2.03) and 2.24 (1.55 to 3.24), depression/anxiety 1.16 (0.96 to 1.41) and 2.27 (1.47 to 3.52).<br>IRR* for EQ VAS (vs 92.5+): 82.5-92.5 – 1.31 (0.93 - 1.84), 72.5-82.5 – 1.31 (0.95 - 1.82), 62.5-72.5 – 1.70 (1.21 - 2.40), 52.5-62.5 – 2.04 (1.41 - 2.97), 42.5-52.5 – 2.26 (1.56 - 3.29), 0-42.5 – 2.04 (1.32 - 3.15).<br>IRR** for EQ-5D overall score: 1 – 1.14 (0.90, 1.43); 2 – 1.30 (1.01, 1.67) 3 – 1.61 (1.23, 2.12); 4+ – 2.25 (1.76, 2.89).<br>Final model – IRR*** for EQ-5D overall score ≥ 3 – RR 1.47 (1.15, 1.88), EQ-5D overall score ≥ 4 – RR 2.06 (1.67, 2.55)	Adjusted,<br>*age, sex, DM, chronic kidney disease, multi-vessel disease, and second prior MI at recruitment, region (fixed effect), and country (using random effect)<br>** age, sex, DM, second prior MI at recruitment, multi-vessel disease, chronic kidney disease, major bleed, PAD, congestive HF, CV enent in past 6 months, on diuretics at enrollment, type of antithrombotic medication, type of management of index MI, EQ-5D overall score (0-10), and region<br>*** age, DM, second prior MI at recruitment, chronic kidney disease, PAD, congestive HF, CV enent in past 6 months, prior major bleed, on diuretics at enrollment, type of management of index MI, EQ-5D overall score (0-10) (7*, 14**, 10***)
Kanwar A, 2020 (Cohort 1), 2020, NA, Oct 2005 - Sep 2008	629	PCI, chronic, inpatient	SF-36, before discharge	Low QOL defined as PCS or MCS score <30	2,9	ACM;<br>Composite (ACM or MI)	HR for ACM or MI for low QoL (<30 for either physical or mental) –1.55 (1.09–2.22); 1.29 (0.89–1.87) when adjusted for frailty.<br>HR for ACM for low QoL (<30 for either physical or mental) –2.37 (1.47–3.82); 1.86 (1.13–3.06) when adjusted for frailty	Adjusted,<br>age, sex, frailty (3)

Supplementary material to 'Associations of health-related quality of life with major adverse cardiovascular and cerebrovascular events for individuals with ischemic heart disease: Systematic review, meta-analysis and evidence mapping'

First author, publication year, study name, recruitment period	N	CAD, enrollment time (after ACS/MI), cohort, settings	HRQoL instrument, assessment time points	HRQoL instrument as a variable in survival analysis	FU, years	Outcome(s)	Associations with outcome(s), Effect estimate (95% confidence interval)	Statistical model, confounders included in the model (their total number)
Nielsen T, 2020, DenHeart survey, Apr 2013 - Apr 2014	260	CABG and PCI, mixed, inpatient	EQ-5D, EQ-VAS, HeartQoL, at discharge or 3 days after	Quartile of the EQ-5D index score; EQ-VAS per 1 point increase HeartQoL Global, physical and emotional per 1 point increase	1	Composite (first event of acute cardiac readmission, revascularisation or ACM)	HR in CABG group: EQ-5D index score (worst quartile) – 1.26 (0.68–2.31), EQ-5D VAS – 1.01 (0.99–1.02), HeartQoL global – 1.05 (0.74–1.50), HeartQoL physical – 1.02 (0.73–1.44), HeartQoL emotional – 1.08 (0.79–1.46).<br><br>HR in PCI group: EQ-5D index score worst quartile – 3.07 (1.67–5.67), EQ-5D VAS - 0.97, 0.96–0.99, HeartQoL global – 0.61 (0.38–0.95), HeartQoL physical - 0.75 (0.51–1.11), HeartQoL emotional - 0.56 (0.39–0.80).	Adjusted,<br><br>sex, age, COPD, prior PCI and current smoking (5)
Pocock S, 2021, TIGRIS, June 2013 - Nov 2014	8978	MI (1-3 years), chronic, outpatient	EQ-5D, EQ-VAS, enrollment (*6 month intervals was not analysed in associations with outcomes)	Categorical EQ-5D score (less than 0.60, 0.60–0.74, 0.75–0.99, 1), categorical EQ-VAS (<60, 60-69, 70-79, 80-89, 90-100), dimentions	2	Composite (CV death, MI, stroke and UA requiring urgent revascularisation)<br>All-cause death	RR for composite end point: categorical EQ-5D (vs 1) <0.6 - 2.31 (1.76 - 3.03), 0.6-0.74 - 1.43 (1.12 - 1.82); EQ-5D domains: mobility 1.14 (0.90 - 1.43), self-care 1.11 (0.84 - 1.36), usual activities 1.30 (1.05 - 1.62), pain/discomfort 1.35 (1.14 - 1.60), anxiety/depression 0.94 (0.78 - 1.14).<br><br>RR for ACM: categorical EQ-5D (vs 1) <0.6 - 3.09 (2.20 - 4.31), 0.6-0.74 – 1.49 (1.07 - 2.07); EQ-5D domains: mobility 1.52 (1.14 - 2.03), self-care 1.70 (1.26 - 2.29), usual activities 1.18 (0.90 - 1.54), pain/discomfort 1.12 (0.90 - 1.39), anxiety/depression 0.91 (0.71 - 1.16).<br><br>The association of EQ-5D and EQ-VAS were considered simultaneously, the index score was independently associated with increased mortality risk (p<0.001), while the VAS score was not (p=0.84).	Adjusted,<br><br>age ≥65 years, DM, second prior MI, chronic kidney disease, HF, PAD, CV event in past 6 months, prior major bleed, medical management only of index MI, on diuretics at enrolment (10)
Ono, 2022, SYNTAXES, Mar 2005 - Apr 2007	1656	PCI and CABG, chronic, inpatient	SF-36, before procedure	PCS and MCS per 10-point increase; terciles of MCS and PCS	10	ACM	HR for 10-point increase in PCS - 0.84 (0.73–0.97), PCS terciles (vs 3rd) 1st - 1.27 (0.91–1.78), 2nd 0.95 (0.68–1.32); 10-point increase in MCS - 0.85 (0.76–	Adjusted,<br><br>age, sex, BMI, medically treated DM, Htn, dyslipidemia, current

Supplementary material to 'Associations of health-related quality of life with major adverse cardiovascular and cerebrovascular events for individuals with ischemic heart disease: Systematic review, meta-analysis and evidence mapping'

First author, publication year, study name, recruitment period	N	CAD, enrollment time (after ACS/MI), cohort, settings	HRQoL instrument, assessment time points	HRQoL instrument as a variable in survival analysis	FU, years	Outcome(s)	Associations with outcome(s), Effect estimate (95% confidence interval)	Statistical model, confounders included in the model (their total number)
							0.95); MCS tercile (vs 3rd) - 1st 1.62 (1.17–2.26), 2nd 1.16 (0.83–1.63)	smoker, previous history of MI, previous history of cerebrovascular disease, PAD, COPD, creatinine clearance, LV EF, left-main CAD involved, and anatomical SYNTAX score, PCS (for MCS) or MCS (for PCS) (16)
Rasmussen, 2022, DenHeart survey, Apr 2013 - Apr 2014	7167	CAD, mixed, inpatient	SF-12, EQ-5D, EQ-VAS, HeartQoL, at discharge or 3 days after	SF-12 PCS, SF-12 MCS, EQ-5D sum, EQ-VAS, HeartQoL	1	ACM	AUC for SF-12 PCS – 0,706, SF-12 MCS – 0,583, EQ-5D sum – 0,639, EQ-VAS – 0,666, HeartQoL – 0,648	Not applicable
Ono, 2022, SYNTAXES, Mar 2005 - Apr 2007	1428	PCI and CABG, chronic, outpatient	SAQ, 6 months and 1 year following revascularisation	Presence of angina by SAQ Angina frequency score at 1 year (≤90 vs >90);<br><br>severity of angina by SAQ Angina frequency score: monthly (70–90), weekly (40–60), or daily (0–30)	9	ACM;<br><br>Components:<br><br>cardiac death;<br><br>MI; stroke; repeat revascularization	SAQ angina frequency score ≤90 vs >90 at 1 year post-revascularisation for outcomes at 5 years: HR for ACM 1.11 (0.83–1.47), CV death 0.92 (0.45–1.91), MI 0.28 (0.08–0.92), stroke 1.03 (0.32–3.29), revascularisation - 1.54 (1.10–2.15).<br><br>SAQ angina frequency score monthly and daily/weekly angina at 1 year post-revascularisation (vs. no-angina): ACM at 10 years – 1.08 (0.79–1.48) and 1.19 (0.71–1.97); HR for revascularisation at 5 years – 1.48 (1.02–2.15) and 1.73 (1.00–2.98).<br><br>Similarly, 6-month angina was not associated with all-cause death (HR 0.91 (0.70–1.20), but was associated with more frequent repeat revascularization at 5 years	Adjusted,<br><br>age, sex, BMI, medically treated DM, Htn, dyslipidaemia, current smokers, previous MI, previous cerebrovascular disease, PAD, COPD, creatinine clearance, LVEF, LM CAD involvement, anatomical SYNTAX score, and achievement of complete revascularization at discharge (16)
Dalsgaard, 2022, DenHeart survey, Apr 2013 - Apr 2014	931	CAD, mixed, inpatient	SF-12, EQ-5D, EQ-VAS HeartQoL, at final discharge or within 3 days after final discharge	Continuous per 1 point increase (SF-12 PCS, SF-12 MCS, EQ-VAS) or decrease (HeartQoL, EQ-5D-5L score)	3	Composite (unplanned, cardiac readmission, revascularization, or ACM)	HR per 1 point decrease: HeartQoL global 0.80 (0.68– 0.95), HeartQoL physical 0.83 (0.71–0.97), HeartQoL emotional 0.88 (0.77, 1.01), EQ-5D-5L Index Score 0.44 (0.20–0.99).	age, Tu co-morbidity index, and BMI (3)

Supplementary material to 'Associations of health-related quality of life with major adverse cardiovascular and cerebrovascular events for individuals with ischemic heart disease: Systematic review, meta-analysis and evidence mapping'

First author, publication year, study name, recruitment period	N	CAD, enrollment time (after ACS/MI), cohort, settings	HRQoL instrument, assessment time points	HRQoL instrument as a variable in survival analysis	FU, years	Outcome(s)	Associations with outcome(s), Effect estimate (95% confidence interval)	Statistical model, confounders included in the model (their total number)
							HR pxer 1 point increase: SF-12 PCS 0.98 (0.97, 1.00), SF-12 MCS 0.98 (0.97, 1.00), EQ-5D VAS 0.99 (0.99, 1.00).	
Bishawi, 2022, ROOBY-FS, 2002-2007	2008	CABG, chronic, inpatient	SAQ, before CABG	Continuous per 1-point improvement in SAQ physical limitation score	5	ACM; CV death	CV death: OR for every 1-point improvement – 0.986, p=0.0116. ACM – OR for every 1-point improvement – 0.989, p=0.0027	Adjusted Age, smoking status, DM, stroke, COPD, PVD, depressed LVEF, high serum creatinine, cardiopulmonary bypass utilization, number of diseased vessels, and history of depression (11)
Bagai, 2022, TIGRIS, June 2013 - Nov 2014	5132	MI, chronic, outpatient	EQ-5D, 1-3 years after MI	Functional impairment summary score calculated using presence of problem with EQ-5D domains—mobility, self-care, and usual activities	2	Composite (CV death, MI, stroke, and unstable angina)	RRs per one-step increase in functional impairment – 1.28 (95% CI: 1.14−1.43) for the CV composite, 1.52 (95% CI: 1.29−1.79) for CV death	Adjusted Age ≥75 years and number of comorbidities (2)
Vyshnevska, 2023, NA, Jan 2020 - Aug 2021	88	MI, acute, inpatient	EQ-VAS, at baseline (12 hours after the onset of first symptoms) and at 6 months	EQ-VAS continuous, per 1 point increase	1	Composite (ACM, MI, ischemic stroke or transient ischemic attack, HF decompensation, hospitalization due to any CV disease deterioration	OR per point increase – 0.89 (0.83- 0.97), p=0.0079	Adjusted Matrix metalloproteinase-9, DM, Htn (3)

Supplementary material to ‘Associations of health-related quality of life with major adverse cardiovascular and cerebrovascular events for individuals with ischemic heart disease: Systematic review, meta-analysis and evidence mapping’

Supplementary Table 3. Risk of bias assessment

Year of publication	First author	Representativeness of the exposed cohort (total sample is more than 1000 and/or consequitive inclusion without specific inclusion or exclusion criteria)	Selection of the non-exposed cohort (from the the same community as the exposed cohort)	Ascertain-ment of exposure	Outcome of interest not present at baseline	Comparability of cohorts (* – adjustment for age and sex only; ** – adjustment for other factors)	Assessment of outcome (independent blind or record linkage)	Follow-up long enough (1 year at least)	Adequacy of follow-up of cohorts (not more than 20% of lost to follow-up)	Total	Overall judgement on the study quality
1998	Lim		*	*	*	**	*	*		7	High
1998	Herlitz	*	*	*	*	**		*		7	Low
1999	Rumsfeld	*	*	*	*	**	*		*	8	High
1999	Bosworth	*	*	*	*	*		*		6	Low
2002	Spertus	*	*	*	*	*	*	*	*	8	High
2003	Mozaffarian	*	*	*	*	**	*	*	*	9	High
2004	Soto	*	*	*	*	**	*	*	*	9	High
2005	Ho		*	*	*	**	*		*	9	High
2007	Kosiborod	*	*	*	*	**	*	*	*	9	High
2007	Pedersen		*	*	*	**		*	*	7	High
2007	Piotrowicz	*	*	*	*	**		*		7	Low
2007	Lenzen	*	*	*	*	**		*	*	8	High
2008	Thombs		*	*	*	**		*	*	7	High
2009	Berecki-Gisolf		*	*	*	**	*	*		7	High
2009	Arnold	*	*	*	*	**		*	*	8	High
2010	Parakh		*	*	*	**	*	*	*	8	High
2010	Norekvål		*	*	*	**	*	*	*	8	High
2010	Schenkeveld	*	*	*	*	**	*	*		7	Low
2011	Kurdyak	*	*	*	*	**	*	*		8	High
2011	Pedersen	*	*	*	*	**	*	*	*	8	High
2011	Singh		*	*	*	**	*	*	*	8	High
2012	Ter Horst	*	*	*	*					4	Low
2012	Grool	*	*	*	*	**		*		7	Low
2013	Nielsen		*	*	*	**	*	*	*	8	High
2014	Tang	*	*	*	*	**		*	*	8	High
2014	Beatty	*	*	*	*	**		*	*	8	High

Supplementary material to 'Associations of health-related quality of life with major adverse cardiovascular and cerebrovascular events for individuals with ischemic heart disease: Systematic review, meta-analysis and evidence mapping'

Year of publication	First author	Representativeness of the exposed cohort (total sample is more than 1000 and/or consequitive inclusion without specific inclusion or exclusion criteria)	Selection of the non-exposed cohort (from the the same community as the exposed cohort)	Ascertain-ment of exposure	Outcome of interest not present at baseline	Comparability of cohorts (* – adjustment for age and sex only; ** – adjustment for other factors)	Assessment of outcome (independent blind or record linkage)	Follow-up long enough (1 year at least)	Adequacy of follow-up of cohorts (not more than 20% of lost to follow-up)	Total	Overall judgement on the study quality
2014	Hofer		*	*	*	**		*	*	7	High
2014	Gunn		*	*	*	**	*	*	*	8	High
2015	Pocock	*	*	*	*	**		*	*	8	High
2015	Moretti		*	*	*	**		*	*	7	High
2015	Hansen		*	*	*	**	*	*	*	8	High
2016	de Jager	*	*	*	*	**	*	*	*	9	High
2017	Compostella		*	*	*	**		*	*	7	High
2018	Patel	*	*	*	*			*		5	Low
2018	Raymakers		*	*	*	**	*	*	*	8	High
2019	Lissåker	*	*	*	*	**	*	*		8	High
2019	Pocock	*	*	*	*	**		*	*	8	High
2019	Berg	*	*	*	*	**		*	*	8	High
2019	Batty		*	*	*	**		*	*	7	High
2019	Rosello	*	*	*	*	**		*		7	Low
2020	Nielsen		*	*	*	**	*	*		7	High
2020	Kanwar		*	*	*	*	*	*	*	7	High
2020	Pocock	*	*	*	*	**		*	*	8	High
2021	Pocock	*	*	*	*	**		*	*	8	High
2022	Rasmussen	*	*	*	*	NA#	NA#	*	*	7	High
2022	Ono [SF-36]	*	*	*	*	**	*	*	*	8	High
2022	Ono [SAQ]	*	*	*	*	**	*	*	*	9	High
2022	Dalsgaard		*	*	*	*	*	*	*	7	High
2022	Bishawi	*	*	*	*	**	*	*	*	9	High
2022	Bagai	*	*	*	*	*		*		7	Low
2023	Vyshnevska		*	*	*	*		*		5	Low

the study by Rasmussen et al. was considered as high quality despite the absence of comparison between poor and good HRQoL (this data has been published in a prior report which was accordingly cited in the manuscript (and which was also included in our systematic review).

Supplementary material to 'Associations of health-related quality of life with major adverse cardiovascular and cerebrovascular events for individuals with ischemic heart disease: Systematic review, meta-analysis and evidence mapping'

References

1. Mommersteeg PMC, Denollet J, Spertus JA, et al. Health status as a risk factor in cardiovascular disease: a systematic review of current evidence. American heart journal 2009;**157**(2):208-18.
2. Kavanagh PL, Frater F, Navarro T, et al. Optimizing a literature surveillance strategy to retrieve sound overall prognosis and risk assessment model papers. Journal of the American Medical Informatics Association 2021;**28**(4):766-71.
3. Westin L, Nilstun T, Carlsson R, et al. Patients with ischemic heart disease: Quality of life predicts long-term mortality. http://dxdoiorg/101080/14017430410003903 2009;**39**(1):50-54.
4. McGowan J, Sampson M, Salzwedel DM, et al. PRESS peer review of electronic search strategies: 2015 guideline statement. Journal of clinical epidemiology 2016;**75**:40-46.
5. Chocron S, Etievent JP, Viel JF, et al. Preoperative quality of life as a predictive factor of 3-year survival after open heart operations. The Annals of thoracic surgery 2000;**69**(3):722-27.
6. Curtis LH, Phelps CE, McDermott MP, et al. The value of patient-reported health status in predicting short-term outcomes after coronary artery bypass graft surgery. Medical care 2002;**40**(11):1090-100.
7. Deaton C WWSRJPRZMCK. Patient Perceived Health Status, Hospital Length of Stay, an... : Journal of Cardiovascular Nursing. J Cardiovasc Nurs 1998;**12**(4):62-71.
8. Dixon T, Lim LLY, F Heller R. Quality of lifean index for identifying high-risk cardiac patients. Journal of Clinical Epidemiology 2001;**54**(9):952-60.
9. Koch CG, Li L, Lauer M, et al. Effect of functional health-related quality of life on long-term survival after cardiac surgery. Circulation 2007;**115**(6):692-99.
10. Pocock SJ, Huo Y, Van de Werf F, et al. Predicting two-year mortality from discharge after acute coronary syndrome: An internationally-based risk score. European Heart Journal: Acute Cardiovascular Care 2019;**8**:727-37.
11. Pocock S, Bueno H, Licour M, et al. Predictors of one-year mortality at hospital discharge after acute coronary syndromes: A new risk score from the EPICOR (long-tErm follow uP of antithrombotic management patterns In acute CORonary syndrome patients) study. European Heart Journal: Acute Cardiovascular Care 2015;**4**(6):509-17.
12. Rossello X, Bueno H, Pocock SJ, et al. Predictors of all-cause mortality and ischemic events within and beyond 1 year after an acute coronary syndrome: Results from the EPICOR registry. Clinical Cardiology 2019;**42**:111-19.
13. Berg SK, Thorup CB, Borregaard B, et al. Patient-reported outcomes are independent predictors of one-year mortality and cardiac events across cardiac diagnoses: Findings from the national DenHeart survey. European Journal of Preventive Cardiology 2019;**26**:624-37.
14. Ter Horst R, Markou ALP, Noyez L. Prognostic value of preoperative quality of life on mortality after isolated elective myocardial revascularization. Interactive Cardiovascular and Thoracic Surgery 2012;**15**:651-54.
15. Pocock S, Brieger DB, Owen R, et al. Health-related quality of life 1-3 years post-myocardial infarction: Its impact on prognosis. Open Heart 2021;**8**.
16. Pocock SJ, Brieger D, Gregson J, et al. Predicting risk of cardiovascular events 1 to 3 years post-myocardial infarction using a global registry. Clinical Cardiology 2020;**43**:24-32.

Supplementary material to 'Associations of health-related quality of life with major adverse cardiovascular and cerebrovascular events for individuals with ischemic heart disease: Systematic review, meta-analysis and evidence mapping'

17. Vyshnevska IR, Petyunina OV, Kopytsya MP, et al. THE ROLE OF BIOCHEMICAL MARKERS AND PATIENT-REPORTED OUTCOMES IN PREDICTING COMPOSITE ONE-YEAR ENDPOINT IN ST-SEGMENT ELEVATION MYOCARDIAL INFARCTION. Polski merkuriusz lekarski : organ Polskiego Towarzystwa Lekarskiego 2023;**51**(1):21-29.
18. Dalsgaard JL, Hansen MS, Thrysoee L, et al. Self-reported health and adverse outcomes among women living with symptoms of angina or unspecific chest pain but no diagnosis of obstructive coronary artery disease - findings from the DenHeart study. European journal of cardiovascular nursing 2022.
19. Pedersen SS, Versteeg H, Denollet J, et al. Patient-rated health status predicts prognosis following percutaneous coronary intervention with drug-eluting stenting. Quality of life research : an international journal of quality of life aspects of treatment, care and rehabilitation 2011;**20**:559-67.
20. Gunn JM, Lautamaki AK, Hirvonen J, et al. The prognostic significance of declining health-related quality of life scores at 6 months after coronary artery bypass surgery. Qjm 2014;**107**:369-74.
21. Nielsen SN, Rasmussen TB, Lassen JF, et al. The association between self-reported health status and adverse events: a comparison among coronary artery bypass grafting (CABG) versus percutaneous coronary intervention (PCI). Quality of Life Research 2020;**29**:3017-29.
22. Rasmussen TB, Borregaard B, Palm P, et al. Patient-reported outcomes, sociodemographic and clinical factors are associated with 1-year mortality in patients with ischemic heart disease-findings from the DenHeart cohort study. Quality of Life Research 2022;**31**(2):389-402.
23. Hansen TB, Thygesen LC, Zwisler AD, et al. Self-reported health-related quality of life predicts 5-year mortality and hospital readmissions in patients with ischaemic heart disease. European Journal of Preventive Cardiology 2015;**22**:882-89.
24. Kosiborod M, Soto GE, Jones PG, et al. Identifying heart failure patients at high risk for near-term cardiovascular events with serial health status assessments. Circulation 2007;**115**:1975-81.
25. Soto GE, Jones P, Weintraub WS, et al. Prognostic value of health status in patients with heart failure after acute myocardial infarction. Circulation 2004;**110**:546-51.
26. Bosworth HB, Siegler IC, Brummett BH, et al. The association between self-rated health and mortality in a well-characterized sample of coronary artery disease patients. Medical care 1999;**37**:1226-36.
27. Kurdyak PA, Chong A, Gnam WH, et al. Depression and self-reported functional status: impact on mortality following acute myocardial infarction. Journal of Evaluation in Clinical Practice 2011;**17**(3):444-51.
28. Tang WHW, Topol EJ, Fan Y, et al. Prognostic value of estimated functional capacity incremental to cardiac biomarkers in stable cardiac patients. Journal of the American Heart Association 2014;**3**.
29. Lim LLY, Johnson NA, O'Connell RL, et al. Quality of life and later adverse health outcomes in patients with suspected heart attack. Australian and New Zealand Journal of Public Health 1998;**22**:540-46.
30. Pedersen SS, Martens EJ, Denollet J, et al. Poor health-related quality of life is a predictor of early, but not late, cardiac events after percutaneous coronary intervention. Psychosomatics 2007;**48**:331-37.
